# A widely applicable and cost-effective method for specific RNA–protein complex isolation

**DOI:** 10.1038/s41598-023-34157-0

**Published:** 2023-04-27

**Authors:** Sam Balzarini, Roosje Van Ende, Arnout Voet, Koen Geuten

**Affiliations:** 1 Molecular Biotechnology of Plants and Micro-organisms, KU Leuven, 3001 Leuven, Belgium; 2 Lab of biomolecular modelling and design, KU Leuven, 3001 Leuven, Belgium

**Keywords:** RNA, RNA-binding proteins

## Abstract

Although methodological advances have been made over the past years, a widely applicable, easily scalable and cost-effective procedure that can be routinely used to isolate specific ribonucleoprotein complexes (RNPs) remains elusive. We describe the “Silica-based Acidic Phase Separation (SAPS)-capture” workflow. This versatile method combines previously described techniques in a cost-effective, optimal and widely applicable protocol. The specific RNP isolation procedure is performed on a pre-purified RNP sample instead of cell lysate. This combination of protocols results in an increased RNP/bead ratio and by consequence a reduced experimental cost. To validate the method, the 18S rRNP of *S. cerevisiae* was captured and to illustrate its applicability we isolated the complete repertoire of RNPs in *A. thaliana*. The procedure we describe can provide the community with a powerful tool to advance the study of the ribonome of a specific RNA molecule in any organism or tissue type.

## Introduction

The interplay between proteins and RNA (the ribonome) plays an important functional role in cell biology. Some important processes regulated by conventional RNA-binding proteins (RBPs), such as the regulation of translation and post-translational modifications^1^, have been known for decades. However recent proteome-wide studies revealed hundreds of novel RBPs without classical RNA-binding domains and raised the concept of not only proteins regulating RNA but as well the potential of RNA to regulate protein function^2^. New functions can be attributed to the dynamics of RNA–protein complex (RNPs) formation: the formation of RNP bodies (e.g. stress granules) driven by liquid–liquid phase separation^3^, the potential of long noncoding RNA to scaffold protein complexes^4^, the role of aberrant RBPs in certain diseases^5^ amongst others. With the emerging understanding of the importance of these complexes, the impetus to develop novel techniques to study RNPs increased. RIC was the first RNA-centric method to allow the isolation of the mRNA interactome targeting the RNPs poly-A tail^6,7^. Multiple other techniques such as CARIC^8^, RICK^9^, TRAPP^10^, VIR-CLASP^11^ to isolate a compilation of RNPs have been developed since. Recently, new methods, based on organic phase separation^12–14^, were developed to isolate the whole compendium of RNPs without a selection of certain RNA elements or post-translational modifications. Additionally, instead of targeting a whole set of RNPs, methods to isolate specific RNP complexes also emerged with ChiRP-MS^15^, CHART-MS^16^ and RAP-MS^17^ (for an extensive review of these methods see Van Ende et al.^18^, Gerber et al.^19^). However, despite the currently available set of techniques, to our knowledge only a few interactomes of specific RNA species have been identified^15,17,20–27^. While proven to be successful, we believe that technical and cost limitations of previous procedures prevent them from being routinely used. The application of these protocols to less uniform and thicker ‘irregular’ samples (e.g. multilayer tissues, plant material etc.) remains challenging as well. The common method for multilayer tissues remains formaldehyde cross-linking because it penetrates more deeply though UV cross-linking of RNA–protein complexes is more specific^15^. There is still a need to expand the procedures available and establish a more broadly applicable protocol to cost-effectively isolate a defined interactome of a specific RNA molecule from a tissue of interest. In this paper, we established a strategy to efficiently isolate specific RNP complexes under physiological conditions by combining and applying key steps from previously described methods.

**Figure 1:**
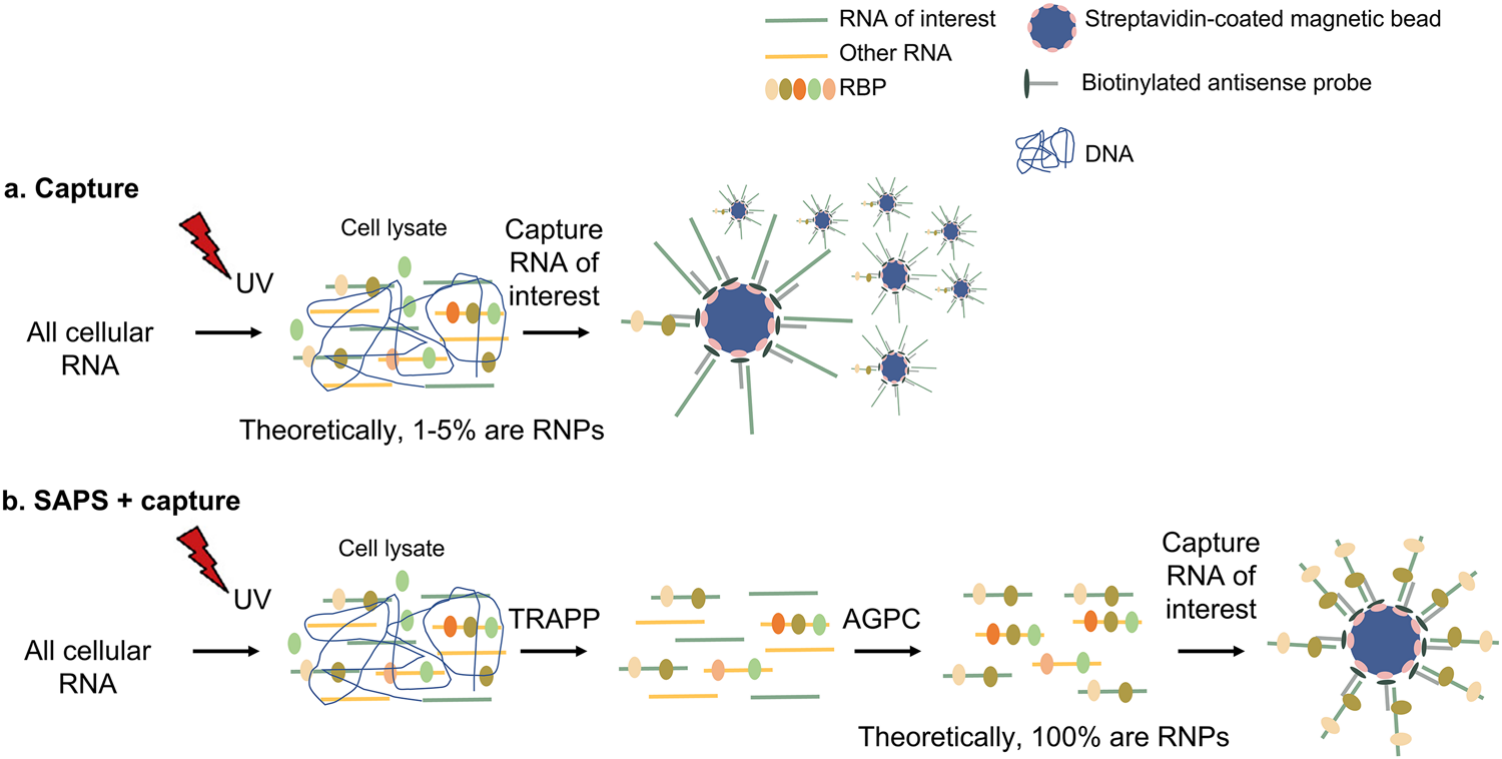
Schematic representing the gain in efficiency when combining a general RNP isolation with a specific RNP capture due to the removal of non-cross-linked RNA. Reworked picture from Van Ende et al. (2020)^18^ (**a**) Most of the previously described specific RNA isolation protocols, capture RNA from the cross-linked lysate. (**b**) If the capture is performed on a pre-purified RNP sample, the protocol will have an increased efficiency of 95–99%.

The gain in efficiency, and by consequence the decrease in experimental costs compared to existing specific RNA isolating protocols, mainly relies on the difference in source material the capture is performed on, which is typically cell lysate^17^. Due to low UV cross-linking efficiency (generally 1–5%)^6,28^, an abundance of non-cross-linked RNA remains present in the cell lysate. When a capture would be performed directly on this lysate, these non-cross-linked molecules will compete with RNPs to hybridize with the probes and given the low cross-linking efficiency, this will drastically decrease the RNP/bead ratio (Fig. 1a). In addition, the presence of naturally biotinylated proteins requires an expensive pre-clearance of the lysate with streptavidin-coated magnetic beads^17^. Our combinatorial protocol, on the other hand, first pre-isolates all UV cross-linked RNPs, the so-called RBPome, by combining a silica-based purification of RNA and RNPs, subsequently followed by an AGPC (acid guanidinium thiocyanate-phenol–chloroform) extraction, further separating the RNP complexes (together referred to as silica-based acidic phase separation or SAPS). The SAPS purification is an alternative yet comparable method to other AGPC procedures, such as XRNAX^14^. The pre-purified RBPome is used as an improved starting point to isolate a specific RNP. By including this general RNP isolation preceding the specific RNP isolation, the absence of non-cross-linked RNA increases the efficiency of the protocol by 95–99% and therefore decreases the cost considerably, as fewer beads are required^18^ (Fig. 1b). By the novel combination of a general RNP isolation with a specific RNP isolation, a more cost-effective protocol was established.

Applying the protocol to the specific isolation of 18S rRNP of *S. cerevisiae* allowed validation, due to the previously identified protein interactors and comparison to the literature^17,29^. We reasoned that once a successful SAPS is established, the RBPome loses its tissue type-dependent character to allow for a universal and streamlined continuation of the RNP isolation procedure. Therefore, to test the wide applicability of the procedure, we also applied the SAPS protocol to whole tissue samples of *Arabidopsis thaliana*, commonly described^30^ as difficult to process. To retain the broad applicability of the combinatorial protocol, methods restricted to cell cultures/systems, such as SILAC labelling or PAR cross-linking, were avoided but can be added to further improve this procedure.

## Results

### Experimental strategy to generalize the procedure

**Figure 2:**
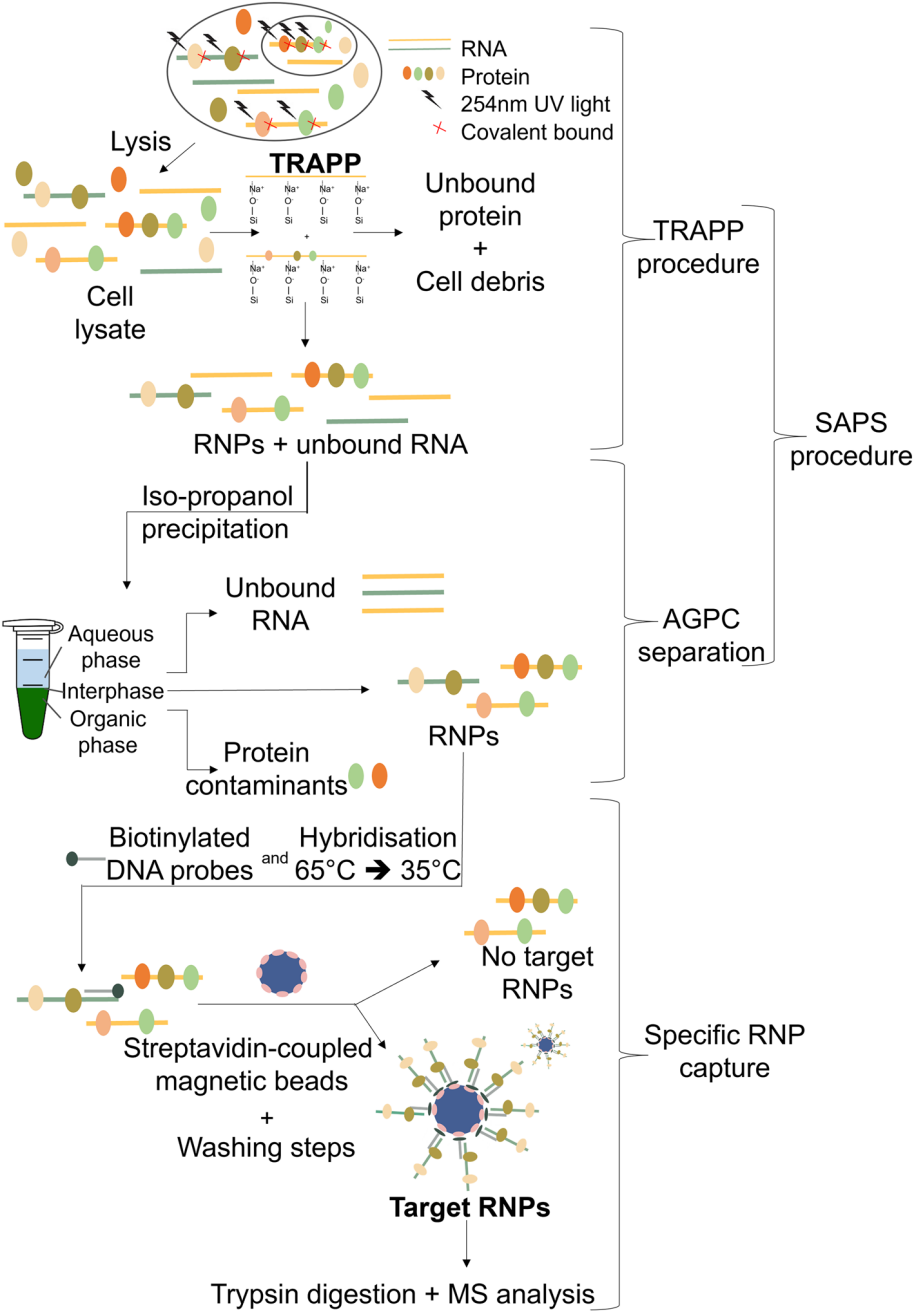
Schematic of the SAPS protocol combined with specific RNP isolation workflow.

We believe some limitations of previous capture procedures prevent them from being commonly used. (1) The difficult nature of cell lysate as a source material to perform an efficient and cost-effective specific RNP isolation procedure on. The presence of RNases/proteinases/secondary metabolites can limit the buffer flexibility and by consequence the applicability of downstream procedures. (2) The limited scalability of the protocol in a cost-effective manner. (3) The partial cross-linking of multilayer tissues due to inefficient penetration of UV light. This can be even more limited by, for example, the presence of a cell wall or UV-absorbing molecules. Figure 2 represents a schematic overview of both the pre-RNP isolation (SAPS) and specific RNP isolation procedure.

### Silica-based acidic phase separation (SAPS) isolating a general set of RNPs

The SAPS procedure can be divided into a solid phase (adapted TRAPP) and AGPC liquid phase separation.

#### Silica pre-purification of non-cross-linked RNA and RNP complexes

In a first step, both non-cross-linked RNA and RNPs were purified according to the protocol (total RNA-associated protein purification (TRAPP)) described by Shchepachev et al.^10^ with minor modifications (described in “Methods”). The TRAPP protocol is a silica-based procedure isolating cross-linked RNP complexes and non-cross-linked RNA molecules clearing the mixture of most non-cross-linked (also naturally biotinylated) proteins, gDNA, lipids and other macromolecules.

#### Isopropanol precipitation and AGPC isolation of RNP complexes

**Figure 3:**
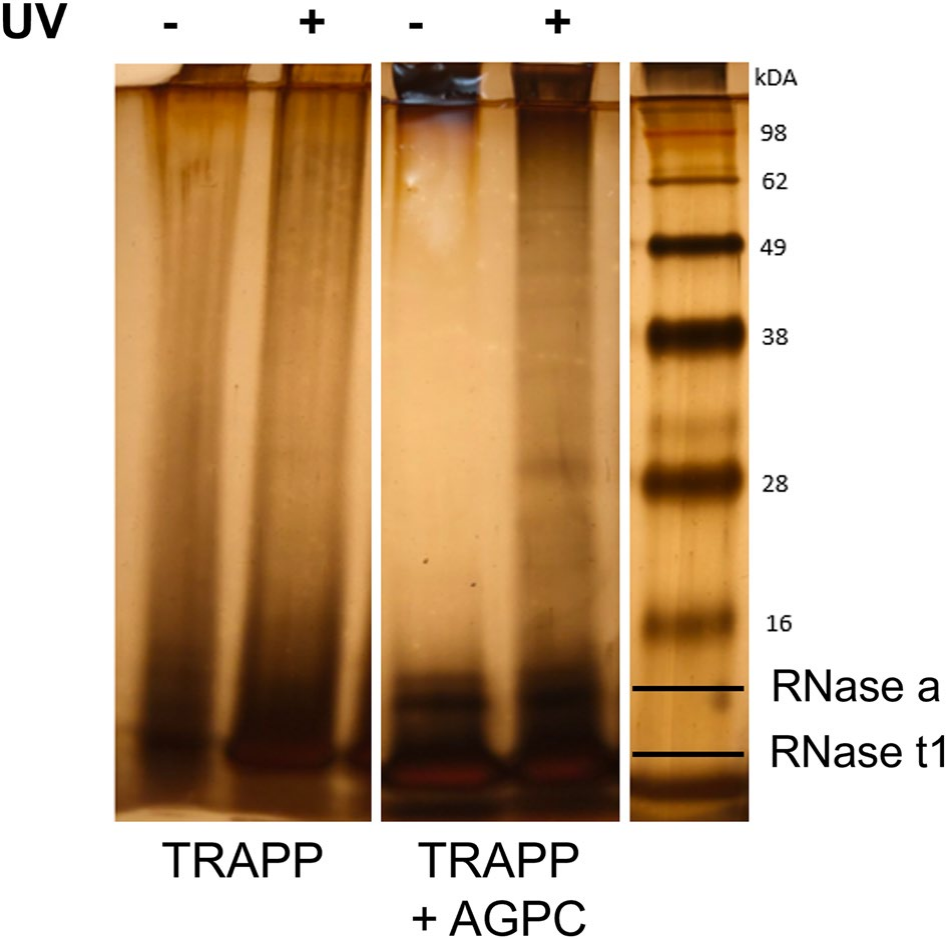
Comparison of non-cross-linked and UV cross-linked sample after TRAPP and after TRAPP + AGPC (= SAPS) visualized by a silver stain assay. *A. thaliana;* 1 J/cm^2^, treated with RNase before loading, 1 g input material. (full size picture in supplementary data Fig. S8).

To concentrate the sample, an isopropanol precipitation is performed after TRAPP. This results in a smaller, more convenient volume for the continuation of the protocol. In the silica-based isolation protocol not only the RNPs are purified, but the non-cross-linked RNA will remain present. These non-cross-linked RNA molecules will also hybridize, potentially even more efficiently, with the complementary probes of the specific RNP capture, resulting in a decreased RNP/bead ratio, as described by Van Ende et al.^18^. To deplete these non-cross-linked RNA ‘contaminants’, a subsequent AGPC isolation is performed. Non-cross-linked RNA will settle in the aqueous phase and RNPs, complexes having both hydrophobic and hydrophilic properties^12–14^, will settle on the interphase. A small amount of non-cross-linked proteins, although stringent washing conditions and chaotropic reagents were used during the TRAPP purification, are still recovered in the non-cross-linked samples^10^. These will settle in the organic phase of a subsequent AGPC purification and by consequence will be depleted as well (Fig. 3). The interphase is isolated and the RNPs are dissolved in a general buffer of choice. This buffer flexibility is enabled by the absence and denaturation of RNases and proteinases during the purification resulting in a stable sample. Therefore, downstream procedures, such as specific RNP capture, can be optimized without a sample-type-origin dependency.

### The pre-purified RBPome as an improved starting point for specific RNP isolation

For a detailed outline of the specific RNP isolation procedure see methods. In short, the purified SAPS sample is mixed with 5 complementary biotinylated DNA oligos (selected based on melting temperature) in a hybridisation buffer. After a stepwise decrease of the temperature to ensure proper annealing, protease-resistant streptavidin-coated magnetic beads are added. After incubation and several washes, the target RNA is eluted. As a negative control, we used scrambled probes.

The isolated RNPs after the SAPS purification provide a uniform/organism-independent input sample for specific RNP capture. This sample is not only depleted of non-cross-linked RNA competing with the complexes for probe hybridization but will be as well free from naturally biotinylated molecules, hybridizing with the streptavidin-coated magnetic beads, avoiding an expensive pre-clearance step.

To validate the SAPS-capture protocol the well-characterized 18S rRNP of *S. cerevisiae* was isolated.

### Quality control (RT-qPCR and BioAnalyzer)

**Figure 4:**
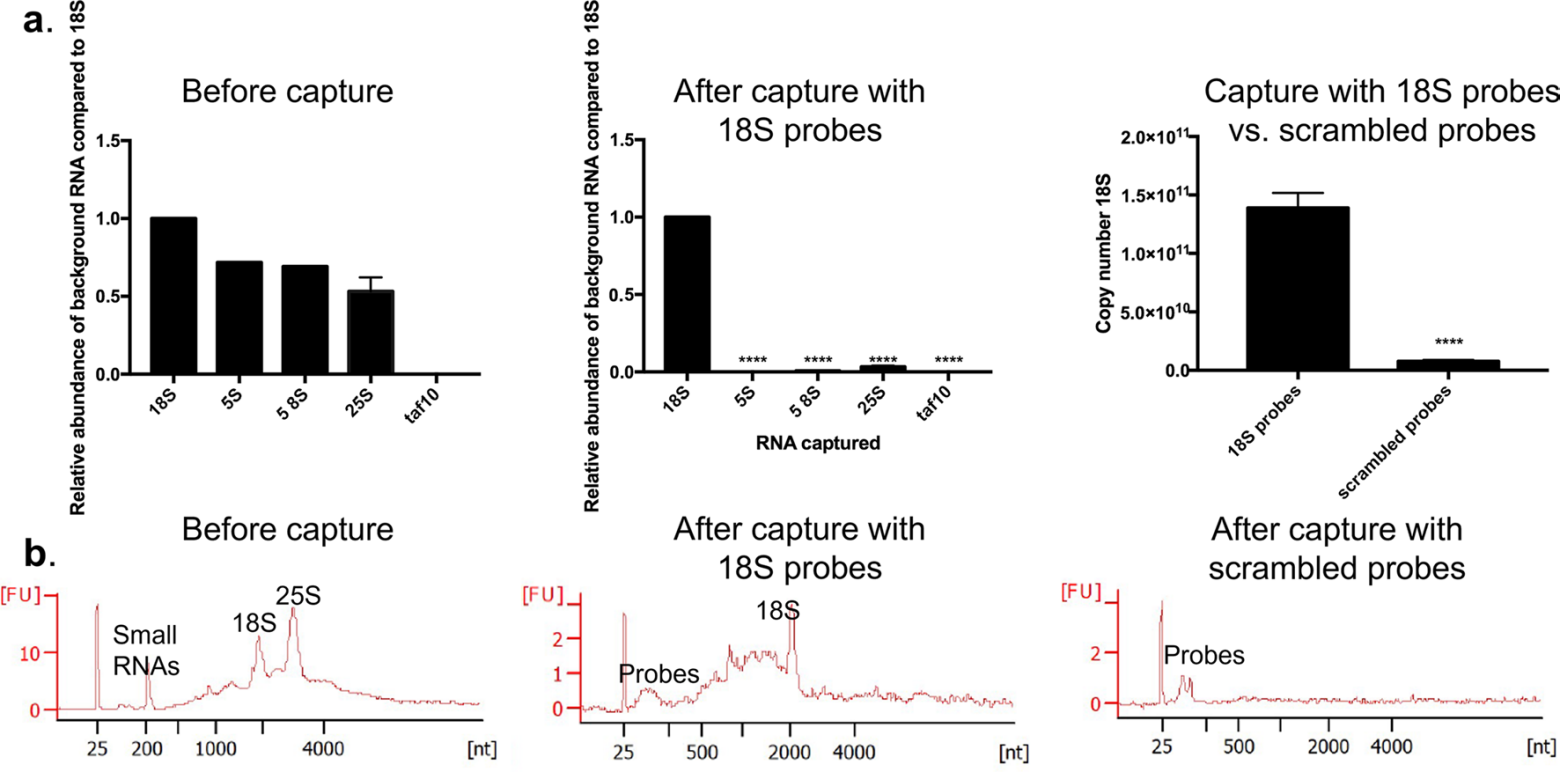
Quality control of 18S rRNA capture (**a**) Quantification of the contamination after capturing 18S. **** represents a two-tailed p-value < 0.0001 (left panel) Relative abundance of 5S rRNA, 5.8S rRNA, 25S rRNA and taf10 compared to 18S rRNA before capture. Values are calculated as the amount of background before capture divided by the amount of 18S before capture. Error bars represent SEM, n = 2. Significance is determined with an unpaired t-test. (middle panel) Relative abundance of 5S rRNA, 5.8S rRNA, 25S rRNA and taf10 compared to 18S rRNA after capture with 18S probes. Values are calculated as the amount of background after capture divided by the amount of 18S after capture. Error bars represent SEM, n = 2. Significance is determined with an unpaired t-test. (right panel) Yield of 18S rRNA comparing captured with 18S probes with capture with scrambled probes. Values are calculated by extrapolation on a standard curve of the plasmid PGEM-T_fulllength18S. Error bars represent SEM, n = 2. Significance is determined with an unpaired t-test. (**b**) RNA pico BioAnalyzer (Agilent) (left panel) SAPS purification diluted to a concentration within the range of an RNA pico chip (middle panel) Capture with 18S probes (right panel) Capture with scrambled probes. FU values are not relevant, due to different elution volumes used when performing the RNP capture for BioAnalyzer vs. for mass spectrometry.

The purity of the SAPS-capture samples was checked with RT-qPCR. Often the validation of this type of protocol is presented as a fold-enrichment. This is a comparison between the ratio of the target RNA and a reference gene before and after the experiment. However, it is possible that, in absolute numbers, the target RNA does not exceed the reference gene concentration, yet is enriched manyfold in comparison to the input^18^. We compared the abundance of 5S rRNA, 5.8S rRNA, 25S rRNA and taf10, an internal control gene^31^, with the abundance of 18S rRNA after the experiment. No ratio compared to the input (= enrichment) was calculated, however for completeness, the expression levels of 25S, 5.8S, 5S, and taf10 before capture are shown (Fig. 4a). After the capture with 18S probes, compared to the abundance of 18S rRNA only 0.1% 5S rRNA, 0.7% 5.8S rRNA, 3% of 25S rRNA and 0, 0001% of taf10 is present. (Fig. 4a). In addition, we compared the amount of 18S rRNA present when the capture was performed with the probes specifically targeting this molecule or when performed with the scrambled probes. There appeared to be 6% 18S rRNA present in the control sample compared to the sample with the specific probes (Fig. 4a). This data indicates the highly specific nature of the protocol.

To address the potential non-specific cross-hybridization of RNA molecules not included in RT-qPCR, a Bioanalyzer assay was performed. It is shown that after the RNA-targeting experiment, the target 18S rRNA is strongly enriched compared to the input and the other abundant RNAs (small RNAs and 25S rRNA) are depleted. The control sample is as well completely depleted (Fig. 4b). As shown by Beckmann et al. (2015), the partial degradation as seen after the capture can be attributed to UV cross-linking^32^. For comparison, a non-cross-linked sample after capture with 18S probes is shown in Supplementary Fig. S1. In Supplementary Fig. S2, we observed this decrease in RNA integrity as a consequence of the cross-linking as well. Besides the UV damage, this partial degradation is also caused by the elution of the complexes at 95 degrees. This is circumvented in the final protocol by on-bead trypsin digestion.

### LC–MS/MS analysis 18S rRNA interactome

A set of 54 proteins (Supplementary Table 1) was significantly enriched either quantitatively (adjusted p-value < 0.01; |log2FC|> 2) or semi-quantitatively (no value for the scrambled probes and a value for at least four out of five replicates for the 18S probes) for the 18S rRNA interactome when comparing with a capture with scrambled probes.

#### Ribosomal proteins

- Proteins of the small ribosomal subunit

**Figure 5:**
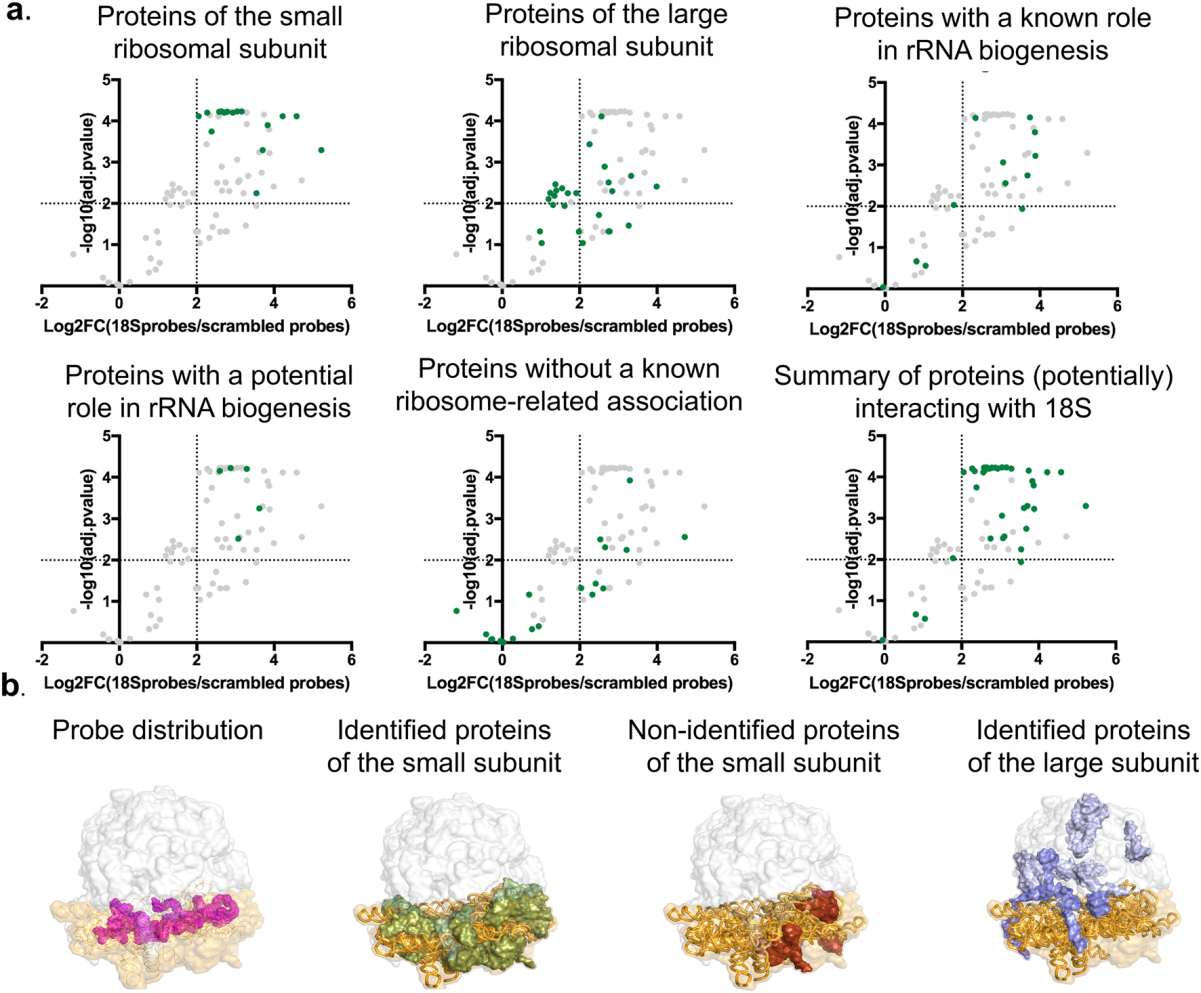
Visualization of different protein groups identified in 18S rRNA interactome (**a**) on volcano plot (in green). Significant proteins are situated in the upper right quadrants (adjusted p-value < 0.01; |log2FC|> 2). From left to right, upper panels: proteins of the small ribosomal subunit, proteins of the large ribosomal subunit, proteins with a role in rRNA biogenesis. From left to right, lower panel: proteins with a potential role in rRNA biogenesis, proteins without known ribosome-related association, summary of proteins (potentially) interacting with 18S. (**b**) CryoEM structure of the yeast ribosome, In orange: 18S rRNA, white shades: large ribosomal subunit, orange shades: small ribosomal subunit. From left to right: Distribution of the probes along 18S rRNA, Significantly enriched proteins of the small ribosomal subunit, Proteins of the small ribosomal subunit not significantly enriched in our dataset, Significantly enriched proteins of the large subunit. In dark blue, the proteins interacting with the 18S rRNA.

22 of the 33 (66%) proteins of the small subunit (40S with 18S rRNA as a scaffold) were identified (Fig. 5a). There are multiple possible explanations for only enriching a subset of all ribosomal proteins of the small subunit. Inefficient UV cross-linking of proteins can occur when the protein interacts with dsRNA stretches^2,29^. Additionally, smaller proteins have fewer chances of being identified due to a lower number of peptides injected into the mass spectrometer. We indeed observed a bias towards identifying mainly larger proteins (Supplementary Table 2). Finally, proteins with only a few direct contacts with the RNA will also result in poor UV cross-linking proteins. With the identification of 66% of all proteins of the small subunit, we validated our approach to perform equally as good as RAP-MS^17^. When isolating 18S rRNA in human cells, Mchugh et al. (2015) identified 21 of the 31 (67%) small ribosomal proteins. Using LNA/DNA mixmer probes^29^, 10% of the small ribosomal proteins were identified.

- Ribosomal proteins of the large subunit

10 of the 54 significantly enriched proteins are proteins known to be a part of the large ribosomal subunit (60S with 25S rRNA as a scaffold) (Fig. 5a). Three of these proteins, RPL19, RPL24 and RPL30, are known to form eukaryotic specific intersubunit bridges to establish the 80S ribosome. RPL19 will interact through its C-terminal α-helical domain with expansion segment 6 of the 18S rRNA (and additionally some small ribosomal subunit proteins: RPS7 and RPS17) forming the eB12 bridge^33,34^. The eB13 bridge is formed by RPL24 interacting with h6, h10 and h44 of 18S rRNA through the linker and α-helix (and additionally a small ribosomal subunit protein: RPS6)^33,34^. RPL30 will form the intersubunit bridge eB9 by interacting with h22 of the 18S rRNA^35^. All three are most likely co-purified due to their physical interaction with 18S rRNA and not due to their protein–protein interaction with 40S ribosomal proteins (RPS6, RPS7 and RPS17; all enriched in the 18S rRNA interactome). Evidence for this is provided by the described intersubunit interaction eB1b between RPS18 and RPL11. RPS18 is found in our dataset, whereas its protein interactor RPL11 is not^36^.

#### Non-ribosomal proteins

22 of the 54 proteins, enriched in the 18S rRNA interactome, are non-ribosomal proteins.

- Proteins with a (potential) role in rRNA biogenesis

11 of these 22 non-ribosomal proteins are known to play a role in rRNA biogenesis (Fig. 5a). These include, identified by quantitative analysis, YEF3/HEF3, NPL3, NSR1, TIF11, NIP1, EFT1, TIF1. In short, YEF3/HEF3 a ribosome-dependent ATPase is also known to physically interact with the 18S rRNA^37^. NPL3 is necessary for pre-rRNA splicing^38^ and is co-transcriptionally loaded to the 5’end of the rRNA^39^. NSR1 is involved in pre-rRNA processing and small subunit assembly. This protein is structurally similar to nucleolin, a mammalian protein known to associate with pre-rRNA^40^. TIF11 plays a role in ribosome dissociation and is thought to interact with the phosphate backbone of 16S in prokaryotes^41^. NIP1 is a protein of the multifactor eIF3 and plays a role in connecting methionine tRNAi to the 40S ribosome. Physical interaction between NIP1 and 18S rRNA is previously shown^42^. EFT1 plays a role in ribosomal translocation during protein synthesis and is a known interactor of 18S rRNA^43^. TIF1 is involved in translation initiation^44^. By semi-quantitative analysis, NOP58, RLP7, NOP56 and TIF4631 were identified. NOP58 and NOP56 are known to function in ribosome biogenesis^45^. RLP7 is involved in 25S pre-RNA processing, but some articles report background interaction with 18S rRNA as well^46,47^. Lastly, TIF4631 is as well labelled as an RBP essential for translation initiation^48,49^.

Five out of the 22 non-ribosomal proteins significantly enriched proteins in the 18S rRNA interactome (Fig. 5a) are not yet described to physically interact with 18S rRNA but interestingly, the literature suggests a potential link with the ribosomal small subunit. In short, BFR1 and SCP160 often co-purify with polysomes, also suggesting a role in translation^50,51^. SBP1 is known to play a role in translation inhibition of PAB1 by a not yet fully elucidated mechanism^52^. PAB1 plays a key role in translation initiation^53^. Lastly, PUB1 is involved in translation termination through interaction with eRF3, however, this interaction could not be functionally validated, suggesting other mechanisms/interactions to be involved as well^54^.

- Proteins without known ribosome-related association

Six of the 22 non-ribosomal proteins appear to not have a link with the small ribosomal subunit (Fig. 5a). These proteins include, identified by quantitative analysis, CDC19/PYK2, PMA1/PMA2, TDH2;TDH3;TDH1, MBF1 and a putative uncharacterized protein. By semi-quantitative analysis, additionally, PGK1 was identified. These proteins can be either contamination or not yet described to be functional in rRNA biogenesis.

To conclude, 22 of the 54 (quantitative and semi-quantitative) proteins (40.7%) were identified as ribosomal proteins of the small subunit. 10 out of the 54 proteins (18.5%) are identified as ribosomal proteins of the large subunit, of which three (5.5%) are known to directly interact with 18S rRNA. 11 of the 54 proteins (20.4%) are non-ribosomal proteins with a known function in rRNA biogenesis. Five out of 54 proteins (9.3%) are proteins with a potential link to rRNA biogenesis and lastly, six out of 54 (11.1%) proteins do not have a known role in rRNA life. In total 75.9% of all significantly enriched proteins of the 18S rRNA interactome appear to be (potential) interactors of the 18S rRNA (Fig. 5a). For comparison, using the RAP-MS protocol^17^ 105 proteins were found to be significantly enriched. 98 (i.e. 93%) were labelled as ribosomal proteins, involved in ribosomal processing, involved in ribosome assembly, translational regulators and ribosome interactors. The numerical difference of significantly enriched proteins (105 vs. 54) between both experiments can be explained by the more stringent cut-off we used (fold change of 3 vs. fold change of 4). Additionally, in the top 15 proteins of the RAP-MS approach, 3 proteins of the large subunit were significantly enriched. However, these proteins were labelled as being within the 98 protein interactors, whereas in our dataset these were labelled as contaminants.

We believe that substituting cell lysate as a source material for a pre-purified RNP sample is the strength of our protocol. This results in a procedure which is highly cost-effective, enabling research labs to perform this type of experiment on a larger scale.

#### CryoEM structure

To inspect for biases and correlations, we visualized the significantly enriched ribosomal proteins in the cryoEM structure (PDB entry: 3JJ7) of this complex. Figure 5b (first panel) shows the probe distribution along the 18S rRNA. Figure 5b (second panel) pictures all 22 identified proteins of the small ribosomal subunit. Figure 5b (third panel) visualizes the ribosomal proteins of the small subunit that were not enriched in our dataset. Besides size and protein-RNA contact sites, protein localization appears to contribute to not being identified, as these proteins are grouped. However, we don’t see an immediate explanation for this. Figure 5b (fourth panel) shows the enriched ribosomal proteins of the large subunit, with in dark blue the proteins interacting with 18S rRNA. Seven proteins of the large ribosomal subunit are likely to be contaminants of the protocol. If studying p-values, it is clear that these contaminants have larger p-values but remain still significant. A more stringent cut-off for example p < 0.001 would result in four of the seven ribosomal protein contaminants becoming not significant. In addition, four of the six proteins without a described ribosome-related association would be as well labelled as not significant. Only one of the 22 proteins of the small subunit and one intersubunit bridging protein would not be enriched if using this more stringent analysis. Alternatively, instead of using stringent cut-offs, analyzing more replicates could contribute to even more clear discrimination between interactors and contaminants.

### Key optimizations for the establishment of the protocol

Four additional optimizations, that improve the protocol greatly were (1) performing a DNase treatment after TRAPP, (2) optimization of the probes/beads ratio for to the input material, resulting in the highest yield and lowest contamination of 25S rRNA after capture with 18S probes. Adding the correct amount of beads is not only cost-effective but will also decrease the peak of unnecessary streptavidin peptides in mass spectrometry. What also contributed to a decreased amount of streptavidin is (3) the use of protease-resistant streptavidin-coated magnetic beads^55^, which additionally avoids the use of heat or benzonase treatment to elute the proteins. (4) Adding formamide to the wash buffers of the RNP capture to enhance RNA integrity and capture specificity.

#### DNase treatment

Although both the washing steps and the acidic pH of the TRAPP protocol are designed to reduce the recovery of DNA, it was shown that DNA is still partially present in the eluate^10^. Evidence for the presence of remaining DNA after SAPS is the high qPCR signal for a non-reverse-transcribed sample. After DNase treatment, qPCR analysis of the non-reverse-transcribed sample approached the values of the non-template control. Additionally, gel electrophoresis confirms the presence of DNA after the silica-based purification (Supplementary Fig. S3).

It is often assumed that DNA UV cross-links less efficient with their protein interaction partners. This is a consequence of the preference of amino acids to cross-link with nucleotide bases instead of the phosphate backbone, in particular with uracil only present in the RNA^56^. However, recently Stützer et al. (2020) have shown efficient UV_254nm_ cross-linking of DNA–protein interactions as well^57^.

When we consider the low efficiency of the UV cross-linking procedure, only 1–5% of the total RNA will be covalently linked to its interacting proteins and by consequence migrate to the interphase. DNA, however less abundant in the cell, will completely (cross-linked or not) settle in the interphase. For this reason, the fraction of DNA in the interphase can be a substantial amount compared to the RNA in the interphase and interfere with the capture. For low-abundance species, the impact of the tendency of DNA to precipitate in the interphase will potentially be even larger. For the samples prepared for specific RNA-targeting, the DNA (cross-linked or not) would occupy the probes and thereby decrease the RNP/bead ratio. Additionally, the DNA-binding proteins would be significantly enriched in the cross-linked samples resulting in false positives. Therefore, a DNase treatment is required (alternatively intron-spanning probes can be used, optimized sonication or several washes of the interphase).

**Figure 6:**
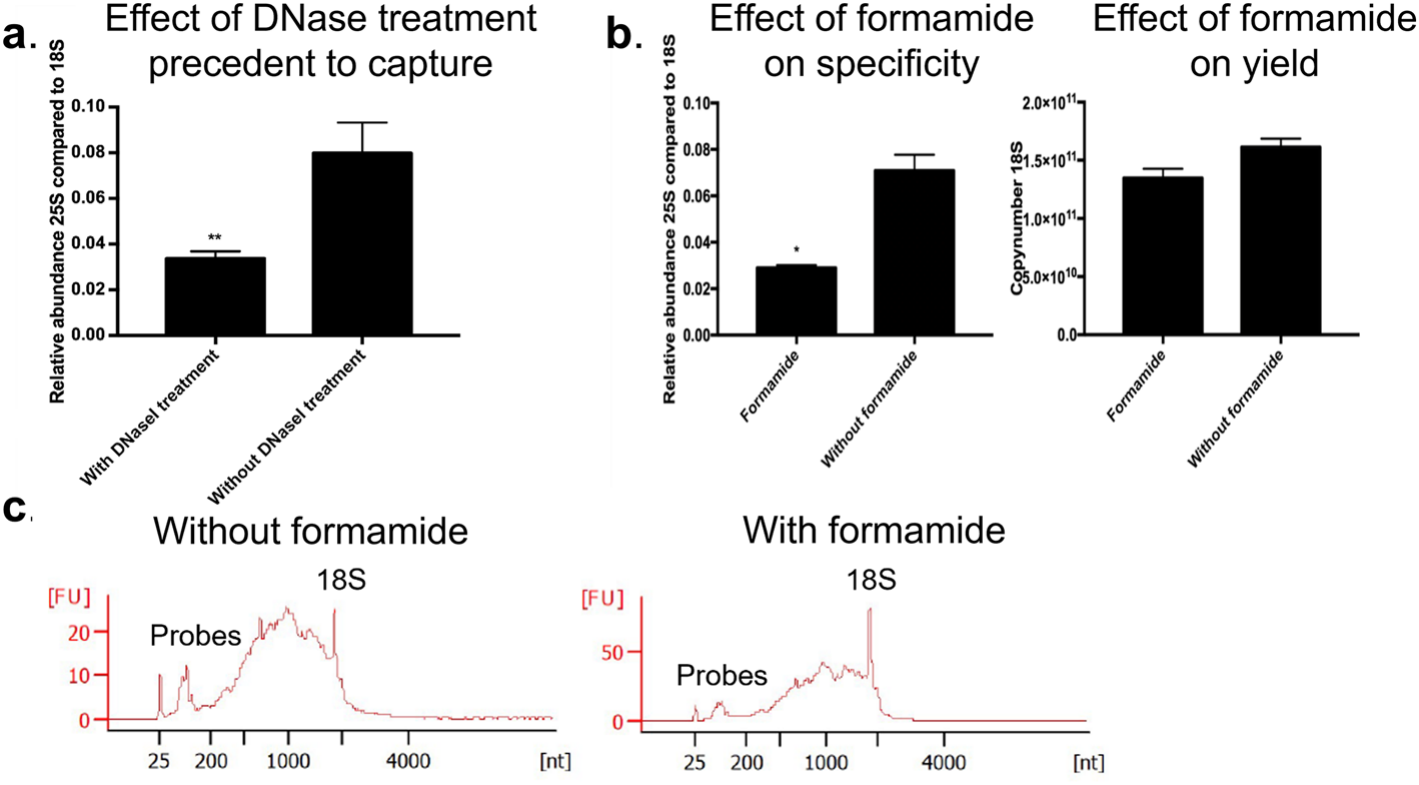
Key optimizations (**a**) Relative abundance of 25S rRNA compared to 18S rRNA after capture with 18S probes comparing with and without DNase treatment. Values are calculated as the amount of background after capture divided by the amount of 18S after capture. ** represents a two-tailed p-value < 0.01. Error bars represent SEM, n = 2. Significance is determined with an unpaired t-test. (**b**) Effect of formamide * represents a two-tailed p-value < 0.05. (Left panel) Relative abundance of 25S compared to 18S with or w/o formamide. Values are calculated as the amount of 25S after capture divided by the amount of 18S after capture. Error bars represent SEM, n = 2. (Right panel) Yield after capture with or w/o formamide. Values are calculated by extrapolation on a standard curve of the plasmid PGEM-T_fulllength18S. Error bars represent SEM, n = 2. Significance is determined with an unpaired t-test. (**c**) Pico BioAnalyzer (Agilent) (Left panel) Capture 18S rRNP w/o formamide (Right panel) Capture 18S rRNP with formamide. FU values are not relevant, due to different elution volumes used when performing the RNP capture for BioAnalyzer vs. for mass spectrometry.

The necessity of performing this enzymatic treatment is shown in Fig. 6a. We performed a specific 18S rRNA isolation with or without a precedent DNase treatment. When no DNase treatment is performed, not only 18S rRNA, but also 18S DNA will be specifically isolated from the SAPS input sample. In *S. cerevisiae* 18S and 25S are both located on chromosome XII (nucleosome) and by consequence, by targeting 18S, 25S DNA will be co-purified. With the DNase treatment included, we noticed indeed a reduced relative abundance of 25S rRNA/DNA contamination in the 18S rRNA interactome, as the purification of 18S rRNA remains equally efficient, but the 25S DNA is not purified anymore.

#### Optimal probes/beads ratio

**Figure 7:**
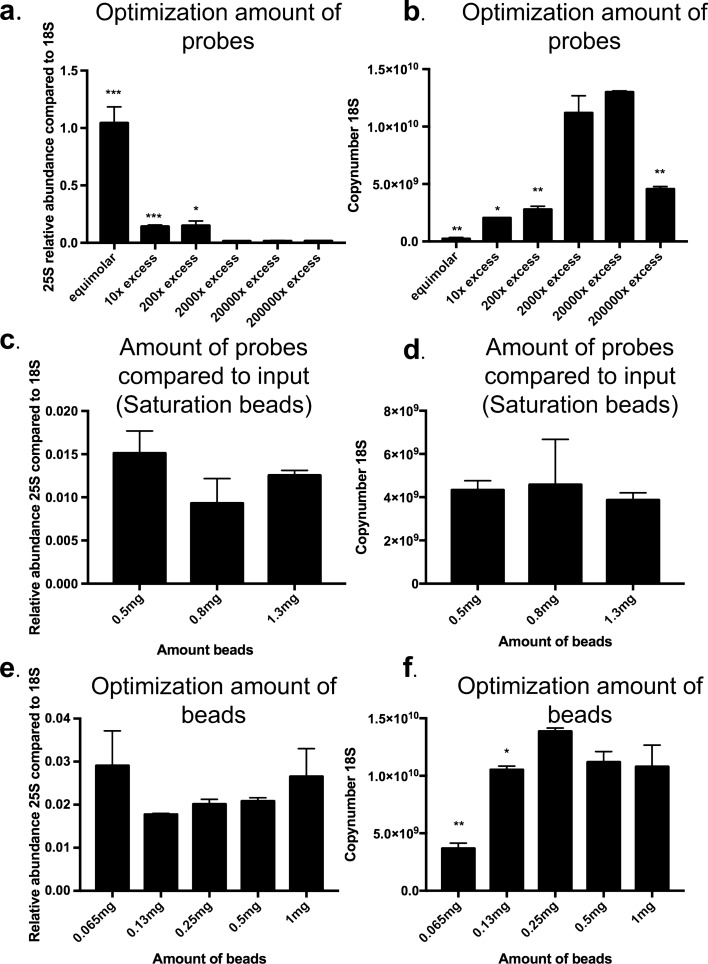
Determining amount of probes and beads required. *** represents a two-tailed p-value < 0.001, ** a two-tailed p-value < 0.01, * a two-tailed p-value < 0.05 (**a**) Relative abundance of 25S compared to 18S for different amount of probes (input 1.5 × 10^9^ and 0.5 mg beads). Values are calculated as the amount of 25S after capture divided by the amount of 18S after capture. Error bars represent SEM, n = 2. (**b**) Yield after capture for a different amount of probes. (input 1.5 × 10^9^ and 0.5 mg beads). Values are calculated by extrapolation on a standard curve of the plasmid PGEM-T_fulllength18S. Error bars represent SEM, n = 2. (**a,b**) Significance is determined with an unpaired t-test compared to 2,000 × excess. (**c**) Relative abundance of 25S compared to 18S for different amount of beads (input 1.5 × 10^9^ and 200,000 × excess probes) Values are calculated as the amount of 25S after capture divided by the amount of 18S after capture. Error bars represent SEM, n = 2. (**d**) Yield after capture for different amount of beads (input 1.5 × 10^9^ and 200,000 × excess probes). Values are calculated by extrapolation on a standard curve of the plasmid PGEM-T_fulllength18S. Error bars represent SEM, n = 2. (**c,d**) Significance is determined with an unpaired t-test compared to 0.5 mg. (**e**) Relative abundance of 25S compared to 18S for different amount of beads (input 1.5 × 10^9^ and 2000 × excess probes). Values are calculated as the amount of 25S after capture divided by the amount of 18S after capture. Error bars represent SEM, n = 2. (**f**) Yield after capture for different amount of beads (input 1.5 × 10^9^ and 2000 × excess probes). Values are calculated by extrapolation on a standard curve of the plasmid PGEM-T_fulllength18S. Error bars represent SEM, n = 2. (**e,f**) Significance is determined with an unpaired t-test compared 0.25 mg.

We determined the optimal probes/beads ratio compared to the copy number of the target in the input sample for specific RNP isolation. We maximized the yield (determined as the copy number of 18S rRNA molecules) in combination with a minimization of background noise (determined by measuring relative levels of 25S rRNA) for the lowest amount of both probes and beads required. A concentration range of the probe mix was tested with a fixed number of beads (0.5 mg) and input (1.5E9 copies of target RNA). The minimal amount of probes required was determined to be an excess of 2,000 compared to the input (Fig. 7a,b). We next investigated whether the reduced yield for the highest amount of probes (an excess of 200,000) was a consequence of the saturation of the streptavidin-coated magnetic beads. As shown in Fig. 7c,d, the yield does not increase with an increasing amount of beads disproving this hypothesis. For the minimal amount of probes (excess of 2000) required, the amount of beads was minimized as well. The lowest amount of streptavidin-coated magnetic beads required appeared to be 0.250 mg. For this amount, the beads are saturated as further increasing the amount of beads did not result in an increased yield (Fig. 7e,f).

#### Formamide

Formamide, which destabilizes hydrogen bonds of nucleic acids, was added to the buffers used for the specific RNP isolation. The melting temperatures could be lowered by 10–20 °C (0.5 °C/%formamide) without losing specificity or yield (Fig. 6b). Aside from the positive effect of lower temperatures (final temperatures being 65 °C to 35 °C for hybridization, 60 °C and 55 °C for washing) on RNA integrity, formamide also destabilizes RNases^58^, which is again contributing to the sample integrity as was observed on BioAnalyzer (Fig. 6c). For the washing steps, the formamide concentration is lowered to 20% for its potentially destabilizing effect on biotin-streptavidin interaction. Commonly this type of protocol is described to be performed at even lower temperatures^17^, however by determining all temperatures based on the buffer composition and the melting temperatures of the probes, more nonspecific binders will be eluted during washing steps. In addition, secondary structures of the RNA molecule will be denatured in the first step, resulting in more efficient binding of the probes and circumventing the need for a preliminary assay to determine secondary structures such as an RNase H assay^59^.

### SAPS protocol for multilayer tissues

Next, we tested the applicability of the protocol to multilayer tissues, namely *A. thaliana* mature leaves. The main goal was to establish a successful SAPS experiment for the continuation to specific RNP isolation being organism-independent. We believe that the sole modifications to the procedure are: (1) The use of an appropriate organism-/tissue-dependent lysis buffer. For *S. cerevisiae*, the lysis buffer described in the TRAPP protocol was used. For *A. thaliana* mature leaves, the plant-specific TRIsure™ lysis buffer was used. (2) The optimization of the UV cross-linking of multilayer tissues, which is generally challenging due to the inefficient penetration of the light. We decided to explore an alternative UV cross-linking procedure to circumvent these tissue dependencies as much as possible. Different doses (1 J/cm^2^ (P1J) and 9 J/cm^2^ (P9J)) of UV cross-linking on frozen powdered tissue which, in a way mimics a cell culture^13^, have been explored and compared with UV cross-linking leaf tissue (0,45 J/cm^2^ (0,45 J) and 9 J/cm^2^ (9 J)). The lower UV doses (P1J/0,45 J) were chosen based on the literature^30,60^, and the higher dose (P9J/9 J) was chosen to test the effect of an unusually high dose. For a detailed outline of the UV cross-linking procedure, see methods.

Except for these two modifications (change of lysis buffer and alternative cross-linking procedure) the SAPS protocol was performed, as described for *S. cerevisiae,* on 5 to 6 weeks old *A. thaliana* plants*.* A non-cross-linked sample was used as a control.

### LC–MS/MS analysis study of the RBPome

#### SAPS reveals a confident RBPome for arabidopsis

**Figure 8:**
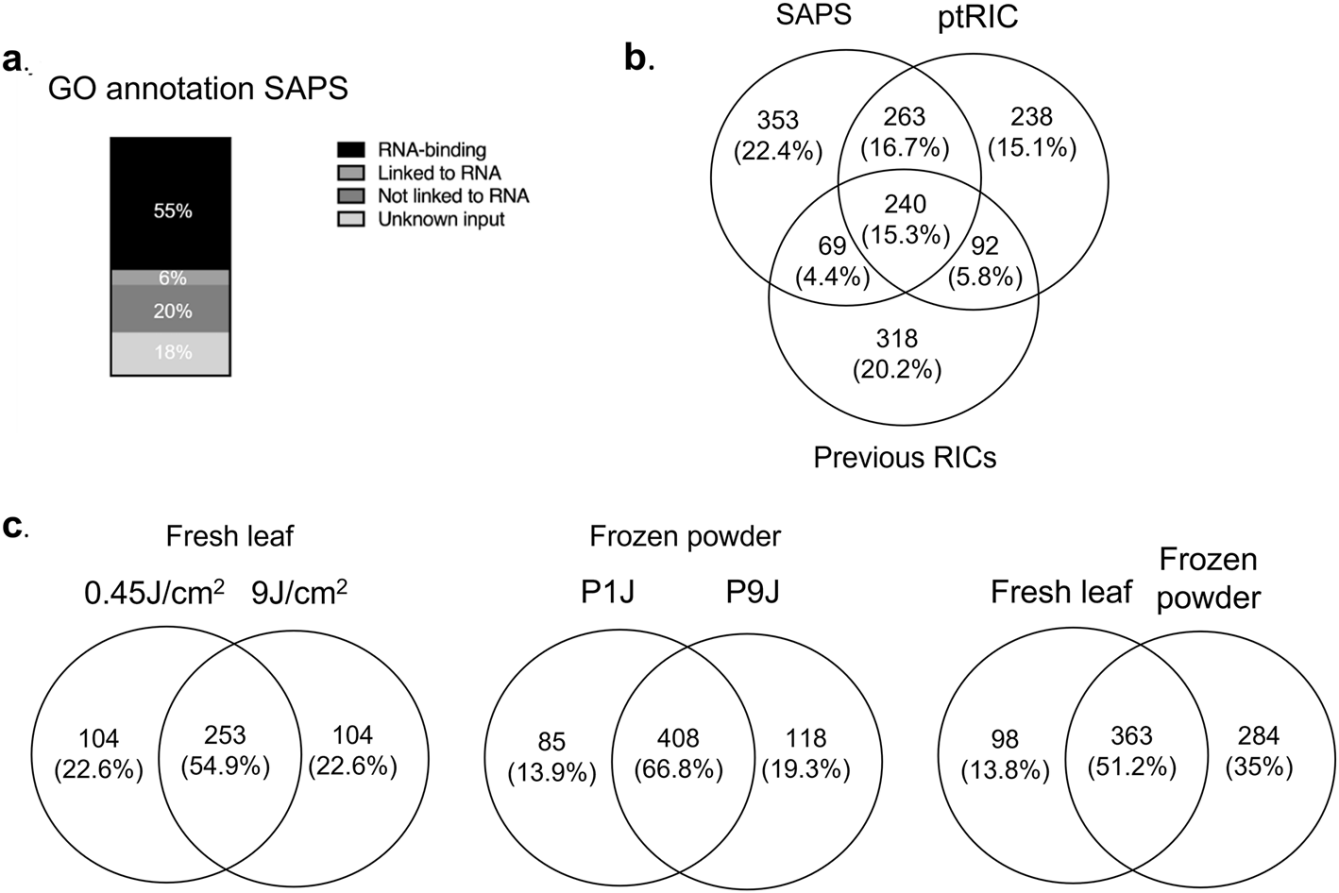
(**a**) An insight into the RBPome of Arabidopsis thaliana leaf tissue isolated by SAPS; the distribution of the RBPome with the GO annotation RNA-binding, linked to RNA, not linked to RNA or proteins with currently unknown functions. (**b**) Comparison of the number of identified RBPs defined by SAPS, ptRIC and previous RIC experiments summarized by Hentze et al.^2^. (**c**) Overlap between the RBPomes of the different sample conditions. The first Venn diagram compared cross-linking conditions (0.45 J and 9 J) on fresh leaves. The second diagram compares UV cross-linking performed on frozen leaf (P1J and P9J). The comparison of fresh leaf and frozen powder conditions is depicted in the third diagram.

To assess the potential of SAPS to purify plant RBPs, a Gene annotation and a Pfam domain analysis have been performed to verify whether an enrichment in RBP terms could be observed. RNA-dependent GO terms are substantially enriched (Fig. 8a) showing that the SAPS isolation protocol targets the RBPs. Approximately 55% of the proteins were annotated as RNA-binding. 6% were linked to RNA activity such as catalytic activity acting on RNA, ribonucleoprotein complex binding and translation factor activity. 20% of the proteins could not be linked to any RNA activity and 18% of the gene ID tags could not be linked to a GO term and therefore could not be assigned to one of the above groups. The range of these numbers is comparable to the previous RIC experiments on multiple organisms^32,61–64^. The proteins of these last two groups could be interesting as previously unknown RNA- interactors. To clarify this, protein-centric approaches such as CLIP could be applied to verify their RNA-binding character. Comparing the sequence and molecular function of newly verified RBPs in these unknown sub-sets could reveal new RNA-binding domains and characteristics of RNA interactors. Additionally, a Pfam study of RNA-binding domains was performed. Approximately, 59% of the SAPS RBPome contain RBDs and 41% domains not linked to RNA (Supplementary Fig. S4).

A comparison of the identified proteins has been made with the ptRIC dataset and all the previous RIC datasets re-analyzed by Hentze et al. (2016) (Fig. 8b). By applying a semi-quantitative and quantitative analysis ptRIC appointed 717 RBP families. Using the same approach SAPS could statistically identify 707 unique RBP families among all the UV conditions combined. These RBP families can harbor multiple protein IDs making comparison over the three datasets rather difficult. Hentze et al. produced a gene locus (gene ID) list of all the previous RICs and therefore both the SAPS and ptRIC dataset was converted to this ID style using uniprot.org for comparison. As can be observed in Fig. 8b, SAPS and ptRIC have a similar percentage overlap with the previous RIC RBP identifications but a significantly higher overlap with each other. This can be explained by the fact that SAPS and ptRIC uses the same plant leaf tissue and growing conditions, clearly different from the previous RIC experiments which used protoplasts, cell suspensions and etiolated seedlings. Therefore, it seems that the identified RBPomes are only a sub-set of the whole RBPome only present during the harvest conditions making comparison of RBPome sets between conditions a powerful tool to identify important RNA-binding regulators.

#### Influence of UV cross-linking conditions on the RBPome

Interestingly, most proteins were identified under the conditions using liquid nitrogen flash-frozen ground powder as source material. This observation suggests that cross-linking frozen powder is more efficient as compared to fresh leaf tissue. This is probably because monolayer cell cultures are mimicked, avoiding the numerous obstructions the UV light has to pass to reach the RNP complexes. If this interpretation is correct, this approach could be a convenient way to study RNP complexes in vivo in theoretically all kinds of difficult to UV cross-link tissues. As was shown recently, significant differences can be observed when studying RBPome dynamics in cell culture instead of tissue^13,65^.

Supplementary Figure S5 shows the distribution of all the identified RBPs between our four conditions. Although the plants used were grown in identical conditions and harvested at the same time, 31% RNA binders unique to one of the conditions could be observed. Similar observations were made by the RBPome studies in multiple organisms summarized by Hentze et al.^2^.

**Figure 9:**
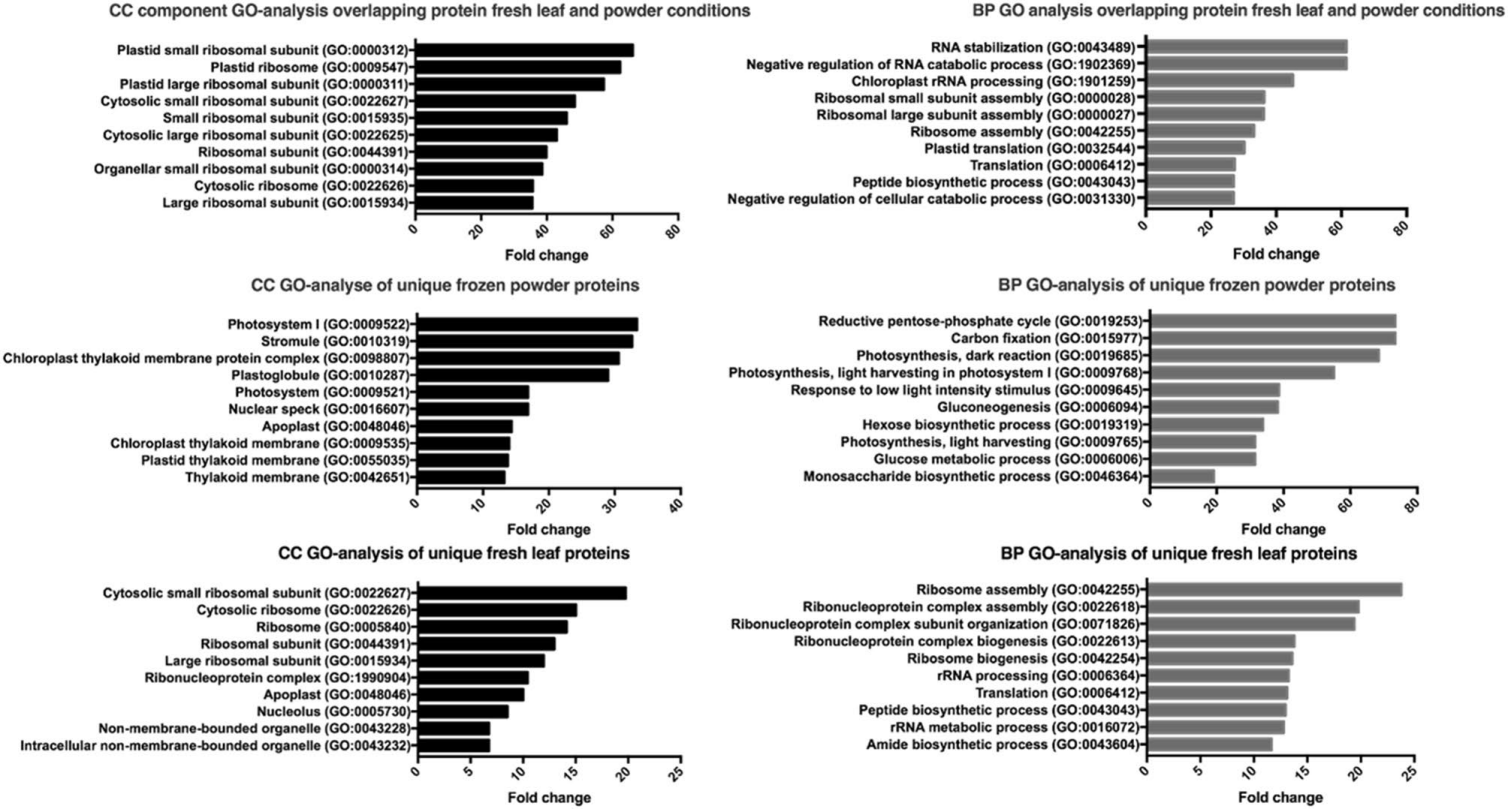
Biological Process (BP) and Cellular Component (CC) GO analysis of the fresh leaf and frozen powder conditions as also their overlap. The 10 highest in terms of enriched fold change are represented for every analysis.

A substantial difference in the number of identified RBPs was observed depending on the UV cross-linking condition (Fig. 8c). Cross-linking of fresh leaf material identified 357 protein families, both for the 0.45 J and 9 J conditions with an overlap of 55% between the two conditions. UV cross-linking performed on frozen leaf powder performed better in terms of quantity (P1J identified 493 and P9J 526 protein families to be RBPs) and consistency with a 67% overlap between both conditions. If fresh leaf and frozen powder conditions were compared an overlap of only 51% is observed. This indicates that the strongest influence on the amount and kind of RBPs as a whole was induced by the sample type (fresh leaves or frozen powder) and to a lesser extent by the UV dose. When a molecular function (MF), biological process (BP) and cellular component (CC) GO-analysis was conducted on both the unique and the overlapping protein IDs of fresh leaf and frozen powder conditions, the following conclusion can be drawn: both the overlapping and unique 84 IDs are highly enriched in MF RNA-binding terms representing the expected RNA-binding character of the protein set (Fig. 9).

The unique IDs of the frozen powder condition are enriched in BP GO terms linked to expressing photosynthesis, gluconeogenesis and carbon fixation resembling healthy cells building up reserves. The BP terms of the fresh leaf condition are more ribosomal, ribonucleoprotein and translationally orientated lacking these photosynthesis and gluconeogenesis terms. This difference could be explained by the fact that fresh leaf material is still living tissue at the moment of UV cross-linking. The intense UV light could provoke rapid changes in the RBPome until the samples are flash-frozen. Although cooled on ice water, the 0.45 J and 9 J conditions were exposed to around 2 min and 25 min of extreme UV irradiation during the cross-linking period, respectively. UV stress can induce upregulation of stress-related pathways and downregulation of photosynthesis-related gene expression^66,67^. This perception of UV light as stress is also apparent from the BP terms in the GO analysis of the fresh leaf conditions. This may result in the dynamic changing of the binding characteristics of the RBPome. UV stress influence could possibly hide interesting RBPome dynamics in the research of RBPs upon a biological cue. In order to perform a successful SAPS-capture on multilayer tissue, based on this experiment analyzing SAPS samples treated with different UV doses, UV cross-linking on frozen powder is recommended.

## Discussion

A key element in establishing a more broadly applicable specific RNP isolation is the optimization of UV cross-linking of multilayer tissues instead of cell cultures. Interestingly, fewer stress-related proteins were identified when compared to the UV cross-linking of fresh leaves, suggesting that a snapshot is taken rather than the effect of a stress cue is observed. To reach deep layers of tissue, we cross-linked liquid nitrogen flash-frozen ground powder of *A. thaliana* leaves, which mimics cross-linking of a monolayer cell culture. The successful isolation of a confident RBPome for *A. thaliana*, a whole tissue sample with a cell wall, suggests that the translation to other tissues and organisms will not provide major difficulties. It appears that the dose of UV determines to a lesser extent the number of RBPs identified. For this reason, it is probably not a requirement to optimize this step for every organism. For the higher UV dose potentially degrading the RNA, the smaller dose is recommended (Supplementary Fig. S6). Once SAPS is performed, the RBPome loses its tissue type-dependent character to allow for a universally applicable continuation of the capture procedure.

We believe that an important strength of our workflow is the pre-isolation of the complete RBPome resulting in a reduced experimental cost. The idea of combining a pre-purification of RNPs with a specific antisense-based RNP isolation is not strictly limited to the combination of techniques of our choice. It can be adjusted to the needs of your experiment. For the pre-purification of the RNPs, we reasoned however the SAPS purification to be the most suitable. The silica-based purification as described by Asencio et al.^68^ and Schepachev et al.^10^ is proven to be a successful strategy when studying the dynamics of the RBPome. However, when the goal is to capture a specific RNP, though the naturally biotinylated proteins are removed through these procedures, the abundance of non-cross-linked RNA remains. Similar reasoning is valid for the tandem RNA isolation procedure (TRIP) combining a RIC pre-purification with a specific antisense-based RNA isolation^69^. The proposed pre-purification depletes biotinylated proteins, ribosomal RNA, reduces sample complexibility etc. however the abundance of non-cross-linked RNA will as well interfere with the RNP/bead ratio. For this reason, an additional/alternative purification step is required and we decided to combine the silica-based purification (TRAPP) with an AGPC separation eliminating non-cross-linked RNA. Such combination was previously described by Trendel et al. (2019) in the XRNAX protocol^14^ but in a reversed order. In their study, an additional purification after the AGPC-based separation was introduced because the presence of true RNA-binding proteins in the interphase of the non-cross-linked sample resulted in no significant enrichment of these in the cross-linked sample. It was hypothesised that some proteins were trapped in the interphase even without cross-linking. The Trendel et al.^14^ approach starts with an AGPC separation followed by a silica-based separation. With the rationale of non-cross-linked proteins being trapped in the interphase, we reasoned that also non-cross-linked RNA could be trapped in the interphase. So while XRNAX could be an alternative method to combine with specific capture, especially when combined with UV cross-linking flash-frozen powder, we chose to reverse the order of AGPC separation and silica-based purification. This is because, as was shown in Fig. 3, in the SAPS protocol the non-cross-linked samples do not retain proteins (and non-cross-linked RNA?) in the interphase, probably due to a less complex TRAPP-purified input sample. In addition, the reverse order is less time-consuming and generates more soluble interphases. An alternative would be to wash the interphase three to four times by repeated AGPC separation to release non-cross-linked proteins and RNA molecules, as described by Queiroz et al. (2019) in the OOPS protocol^12^. However, this procedure as well requires a subsequent clean-up due to the glycosylated proteins, which share physiochemical properties with RNPs, also co-precipitating on the interphase. This is remedied by an RNase treatment of the sample and a final AGPC separation. The released RBPs migrate to the organic phase and by recovering these a pure RBPome is generated. However, due to the RNase treatment, this protocol is also not applicable when a subsequent RNP isolation is aimed for.

In our enriched fraction, 66% of the proteins of the small subunit could be identified. This is in a similar range as the RAP-MS protocol established but with a significant reduction of the experimental cost. If only taking into account the estimated cost of the beads/replicate, considering the beads being the largest expense, for RAP-MS this would be around 2600 euros, for SAPS-capture this would be around 22 euros. A price difference of more than 100x. When taking into account the tiling approach used in RAP-MS, which we did not verify the importance of, and the consequential large amount of required probes, this difference will even be larger.

All currently available methods isolate either overexpression targets or highly abundant targets. For less abundant targets, if the material is not limiting, our workflow can be easily scaled. The sample will be concentrated during the AGPC separation resulting in workable volumes. A good negative control is of great importance. Examples are scrambled probes, RNase-treated samples, non-cross-linked samples, the capture of another RNP, knock-out samples, etc. Generally, a non-cross-linked control is preferred due to probe or target-specific background contamination. However, due to the stringent purification of SAPS before RNA-targeting, these controls appear to be less interesting. We opted for scrambled probes. However, if the experimental set-up allows, this probe-specific contamination can be considered when working with a knock-out sample. For less abundant targets, a combination of negative controls could increase reliability. To increase the probability of detecting low abundant interacting proteins, the background should be as low as possible. An additional Riboclear purification, oligo dT capture or a double capture (Supplementary Fig. S7) might increase the detection of the lowly abundant targets.

To conclude, we applied a cost-effective, widely applicable workflow that first isolates the whole repertoire of RNPs referred to as SAPS. The isolated RBPome can be the starting point for many downstream processes, such as described here, specific RNP isolation. The SAPS-capture workflow was validated for a well-described RNP, namely 18S rRNP of *S. cerevisiae.* Next, the potential of SAPS-capture to be applied to “difficult to handle” samples was validated by investigating SAPS and multiple UV cross-linking procedures using *A. thaliana* mature leaves, where we could isolate a confident RBPome. This indicates the potential of the SAPS-capture workflow to be routinely used because it is both tissue-and organism-independent and cost-effective. Future experiments will validate its applicability to more lowly abundant targets.

## Methods

### Reagents

UVP crosslinker CL-1000: AnalytikJena, USA, 849–30,101-2.

TRIsure™: Meridian BIOSCIENCE, Belgium, BIO-38033.

Nanodrop spectrophotometer ND-1000: Isogen Life Science, The Netherlands, 6211.

DNaseI recombinant, RNase-free: Sigma-Aldrich, Belgium, 4,716,728,001.

Pierce™ silver stain kit: ThermoFisher Scientific, Belgium, 24,612.

Pierce™ BCA protein assay kit: ThermoFisher Scientific, Belgium, 23,225.

Murine RNase inhibitor: New England BioLabs, The Netherlands, M0314S.

SensiFAST SYBR Hi-ROX Kit: GC Biotech, The Netherlands, BIO-92020.

BioAnalyzer 2100: Agilent, Belgium,G2939BA.

Pierce™ Trypsin Protease, MS Grade: ThermoFisher Scientific, Belgium, 90,057.

Streptavidin magnetic beads, New England BioLabs, The Netherlands, S1420S.

OMIX C18 pipette tips: Agilent, Belgium, A57003100.

Benzonase® nuclease: Sigma-Aldrich, Belgium, 70,664–3.

Phoenix Peptide Cleanup Kit: Preomics, Germany, P.O.00023.

Lunatic spectrophotometer: Unchained Labs, USA.

Ultimate™ 3000 RSLCnano system: ThermoFisher Scientific, Belgium, ULTIM3000RSLCNANO.

Nanospray Flex™ Ion Sources: ThermoFisher Scientific, Belgium, ES071.

C18 Reprosil-HD: Dr. Maisch, Germany, r15.b9.

Ultimate™ 3000’s column oven: Thermofisher Scientific, Belgium, 5730.0010.

Silica PicoTip emitter: New Objective, USA, FS360-20–10-N-20-C12.

µPAC™ HPLC Columns: ThermoFisher Scientific, Belgium, COL-NANO200G1B.

Waters nanoEase M/Z HSS T3 Column: Waters Corporation, UK, 186,008,818.

### Biological resources

*Saccharomyces cerevisiae:* S288C.

*Arabidopsis Thaliana*: Colombia-0.

p-GEM^®^-T vector systems: Promega, The Netherlands, A3600.

### Web sites/data base referencing

Probe design: http://array.iis.sinica.edu.tw/ups/index.php.

Genome S288C: https://www.yeastgenome.org/strain/s288c.

Scrambled probe design: https://www.ncbi.nlm.nih.gov/genome/?term=txid12295[Organism:exp].

Blast: https://blast.ncbi.nlm.nih.gov/Blast.cgi.

Calculation melting temperatures: https://sourceforge.net/projects/melting/.

MaxQuant algorithm (version 1.6.17.0 for *A. thaliana*/ version 2.0.1.0 for *S. cerevisiae*).

Protein sequences *A. thaliana*: Swiss-Prot database (database release version of 04_2020).

Protein sequences *S. cerevisiae*: Uniprot database (database release version of 11_2020), https://www.uniprot.org/proteomes/UP000002311.

Perseus software.

R: limma package-moderated t-test.

GO analysis: Panther.

### Preparation of constructs

Plasmid pGEM-T_fulllength18S was generated by amplifying the full-length 18S by primers PCR1_18SF and PCR2_18SR (Supplementary Table 3) from *S. cerevisiae* cDNA and inserted into the vector pGEM-T.

### Yeast growth and UV cross-linking

Yeast cells were grown at 30 °C under shaking (220 rpm) in YPD medium ((w/v) 1% yeast extract, 2% peptone, and 2% D-glucose). The cells were harvested (10 min, 3000 g) for UV cross-linking (254 nm) at mid-log phase OD 0.5–0.6 from 750 mL of media (roughly 5.5 10^9^ cells). The pellet was resuspended in 200 ml ice-cold cross-linking buffer^68^ (25 mM Tris–HCl, pH 7.5; 140 mM NaCl; 1.8 mM MgCl2; and 0.01% NP-40) supplemented with 2% glucose. Per 50 mL, the cells were transferred to a 145/20 mm petri dish and placed on ice in a UVP crosslinker. The cells were irradiated with a dose of 1.2 J/cm^2^. Every 0.4 J/cm^2^, the cells were cooled by swirling for 30 s on ice. After cross-linking, the cells were pelleted (5 min, 3000 g) and frozen in liquid nitrogen.

### Plant growth and UV cross-linking

Five *Arabidopsis thaliana* plants per pot were grown in day-neutral conditions (12 h light, 12 h dark) at 20 °C with a light intensity of 100 µmol/m^2^/s^2^. Different UV conditions were applied to 5–6 weeks old plants. For fresh leaves, doses of 0.45 J/cm^2^ and 9 J/cm^2^ were applied in a UVP crosslinker. For the dose of 9 J/cm^2^, 10 doses of 0.9 J/cm^2^ were administered with short pauses in between to cool the material. The leaves were placed with the abaxial side upwards on icy water and ice was replenished when thawed. After UV cross-linking, the leaves were patted dry and flash-frozen using liquid nitrogen to preserve the RNA–protein molecular interactions and sample integrity. The frozen powder samples were first ground into powder form, mixed with liquid nitrogen and UV cross-linked in a thin layer of powder/liquid nitrogen mixture. Doses of 1 J/cm^2^ (P1J) and 9 J/cm^2^ (P9J) were applied. A maximum of 1 J/cm^2^ was applied during each cross-linking run and extra liquid nitrogen was added when necessary to avoid thawing of the samples. Samples were stored at − 80 °C until further use.

### Silica-based acidic phase separation (SAPS)

The protocol is outlined for 750 mL of yeast cell culture OD 0.5–0.6 or 1 g of plant material but can be easily scaled up/down accordingly.

#### Silica pre-purification of non-cross-linked RNA and RNP complexes

In a first step, both non-cross-linked RNA and RNPs were purified according to the protocol (total RNA-associated protein purification or TRAPP) described by Shchepachev et al.^10^ with minor modifications. In short these include, (1) plant cells were lysed in 10 mL of TRIsure™ supplemented with 10 mM β-mercaptoethanol. The cell lysate was vortexed and incubated for 5 min at RT. (2) All centrifugation steps (both for yeast as plant material) to precipitate cell debris were extended to 15 min at 4,750 g. (3) During the washing steps, silica beads loaded with the RNA and RNPs were precipitated at 2000 g for 2 min. (4) Finally, after elution, the collected eluate was centrifuged for 5 min at maximum speed at 4 °C to remove silica powder remnants, which otherwise interfere with the formation of the interphase. The resulting eluate contains both non-cross-linked RNA and RNP complexes.

#### DNase treatment, isopropanol treatment and AGPC isolation of RNP complexes

For every 10 µg of RNA (measured with Nanodrop spectrophotometer), 1 U of DNaseI, supplemented with DNase incubation buffer, was used. Half of the DNaseI was added and the sample was incubated for 30 min at 37 °C with occasional mixing. Subsequently, the other half was added and incubated for 30 min at 37 °C with occasional mixing.

To remove deoxynucleotides and to concentrate the sample, isopropanol precipitation was performed. The eluate was divided into 750 µl per 2 ml tube. 45 µl of 5 M NaCl and 750 µl of ice-cold isopropanol were added. The solution was cooled and stored overnight at − 20 °C. The RNP and RNA complexes were pelleted by centrifugation at maximum speed for 15 min at 4 °C and washed using 1 ml of 70% ethanol. The pellet was resolubilized into 200 µl of RNase-free water or 10 mM Tris–HCl buffer (pH 7.5) on ice.

To remove both non-cross-linked RNA molecules and remaining protein contaminants, 1.2 ml of Trisure™ was added to every 200 µg of RNA equivalent (measured with Nanodrop spectrophotometer) and mixed vigorously to dissolve all the RNP/RNA molecules properly. If a precipitate was still visible, the mixture was heated to 50 °C and vortexed till everything was dissolved. 250 µl of chloroform was added, vortexed and incubated for 5 min on a rotating mixer. The samples were centrifuged at maximal speed for 15 min at 4 °C to obtain 3 phases. The aqueous phase was removed and the slurry interphase containing the pure RNP complexes was transferred to a new low protein binding tube and dissolved in 200–500 µl 10 mM Tris–HCl RNase-free buffer (pH 7.5). As a quality control, both a silver stain assay and a BCA protein assay were performed. This mixture can be used for the study of the RBPome or as the starting point for the specific RNP-targeting protocol.

### The capture of specific RNP of interest

#### Probe design

Five 60-mer probes with a melting temperature of around 70 °C were designed to specifically target the RNA of interest (Supplementary Table 4). The free software “unique probe selector 2.0” was used. Each DNA oligonucleotide complementary to the RNA sequence of interest was ordered (IDT) with a biotinylated 3’end to enable a capture with streptavidin-coated magnetic beads. For the 18S probes, the RNA sequence of 18S provided by the Saccharomyces Genome Database (SGD) was used. For the scrambled probes, the RNA sequence of Tobacco rattle virus provided by NCBI was used as a template. (These probes were used because of availability in the lab). The scrambled probes were blasted against the genome of *Saccharomyces cerevisiae* to minimize off-targets.

#### RNA-targeting protocol

The protocol is described for one capture. For every replicate, 12 captures were pooled. Generally, one SAPS isolation as described above is sufficient to provide input material for 12 captures (or even more).

1.5 10^9^ copies of the target RNA (determined by absolute RT-qPCR) were mixed with 0.5 mL hybridization buffer (50 mM Tris–HCl pH 7.5, 5 mM EDTA, 500 mM LiCl, 0.2% SDS, 0.1% sodium deoxycholate, 4 M urea) supplemented with 40% deionized formamide, 0.1 mM PMSF, 8 U RNase inhibitor and 0.5 nmol of a mixture of all five probes. This mixture was incubated while shaking (450 rpm) at 65 °C for 10 min. The temperature was slowly lowered to 45 °C, incubated for 5 min and again lowered to 35 °C after which the sample was transferred to ice. 0.25 mg of protease-resistant^55^ streptavidin coated-magnetic beads, which were previously washed 3 times with wash and bind buffer (20 mM Tris–HCl pH 7.5, 500 mM LiCl, 1 mM EDTA), were added together with 0.5 mL hybridization buffer. The probe-RNP complexes were incubated together with the beads for 2 h at 50 °C while shaking (450 rpm). Probe-RNP complexes bound to the beads were then washed for 3 min at 60 °C with wash and bind buffer supplemented with 20% deionized formamide. This step was performed 2 times. Afterwards, the beads were washed for 3 min at 55 °C with low salt buffer (20 mM Tris–HCl pH 7.5, 150 mM LiCl, 1 mM EDTA) supplemented with 20% deionized formamide. The mixture is transferred to a clean low binding tube and a final wash for r 3 min at 55 °C with low salt buffer was conducted. 90 µg beads were removed after the final wash and eluted in 5 µl elution buffer (10 mM Tris–HCl pH 7.5) for 3 min at 95 °C for quality control using RT-qPCR and RNA pico BioAnalyzer. The remaining beads were resolved in 150 µl trypsin digestion buffer (20 mM Tris–HCl pH 8.0, 2 mM CaCl_2_) and incubated for 4 h with 1 µg trypsin at 37˚C. Beads were removed, another 1 µg of trypsin was added and proteins were further digested overnight at 37˚C. Peptides were purified on Omix C18 tips and dried completely in a rotary evaporator. All used binding and washing temperatures were calculated using the free software “MELTING”.

#### Quality control: RT-qPCR and BioAnalyzer

To determine the purity of the specific RNP samples/specificity of the RNA-targeting protocol, both a RT-qPCR and a BioAnalyzer assay were performed to check abundance of non-target genes after the capture according to the protocol of the manufacturer. A standard volume of 7.5 µl was used in the 10 µl reverse transcription reaction volume. The RNA integrity number (BioAnalyzer), which is based on the 25S/18S ratio, could not be determined due to the absence of 25S after the capture. The BioAnalyzer assay was solely performed to check the presence of abundant RNA contaminants following the manufacturing protocol of the RNA 6000 Pico Chip (Agilent).

### Sample preparation for mass spectrometry study of the RBPome in *A. thaliana*

For mass spectrometry sample preparation, 2 g of plant material per replicate were used.

The RNA part of the RNP molecules was degraded using Benzonase 99% pure. The RNP mixture was heated to 80 °C to dissolve liquid–liquid phase separation complexes that can occur and could impede the Benzonase cleaving efficiency. The samples were cooled to 37 °C and 12 U of Benzonase supplemented with 1 mM MgCl_2_ was added. After the Benzonase digest, the SP3 method^70^ was used to digest the proteins into peptides and to desalt the samples following the published protocol scaled to our volumes. The peptides were eluted in 100 µl of 100 mM ammonium bicarbonate (pH 8) and sent on dry ice to the mass spectrometry Facility Core of the University of Ghent for further processing. For each experimental condition of the plant samples, part of one replicate was pre-ran on the mass spectrometry set-up as a trial. The presence of a polymer resulting in clogging of the machine was observed. The samples were further purified using the Phoenix peptide clean-up kit successfully removing the polymer.

### LC–MS/MS analysis

Peptides of the 18S rRNA interactome were re-dissolved in 20 µl loading solvent A (0.1% trifluoroacetic acid in water/acetonitrile (ACN) (98:2, v/v)) of which 2 µl was injected for LC–MS/MS analysis on an Ultimate 3000 RSLCnano system in-line connected to a Q Exactive HF mass spectrometer. Trapping was performed at 10 μl/min for 4 min in loading solvent A on a 20 mm trapping column (made in-house, 100 μm internal diameter (I.D.), 5 μm beads, C18 Reprosil-HD). The peptides were separated on a 250 mm Waters nanoEase M/Z HSS T3 Column, 100 Å, 1.8 µm, 75 µm inner diameter kept at a constant temperature of 45 °C. Peptides were eluted by a non-linear gradient starting at 1% MS solvent B reaching 33% MS solvent B (0.1% FA in water/acetonitrile (2:8, v/v)) in 63 min, 55% MS solvent B (0.1% FA in water/acetonitrile (2:8, v/v)) in 87 min, 99% MS solvent B in 90 min followed by a 10-min wash at 99% MS solvent B and re-equilibration with MS solvent A (0.1% FA in water). The mass spectrometer was operated in data-dependent mode, automatically switching between MS and MS/MS acquisition for the 12 most abundant ion peaks per MS spectrum. Full-scan MS spectra (375–1500 m/z) were acquired at a resolution of 60,000 in the Orbitrap analyzer after accumulation to a target value of 3,000,000. The 12 most intense ions above a threshold value of 15,000 were isolated with a width of 1.5 m/z for fragmentation at a normalized collision energy of 30% after filling the trap at a target value of 100,000 for maximum 80 ms. MS/MS spectra (200–2000 m/z) were acquired at a resolution of 15,000 in the Orbitrap analyzer.

Purified peptides of the plant sample for shotgun analysis were re-dissolved in 20 µl solvent A (0.1% TFA in water/ACN (98:2, v/v) and peptide concentration was determined by measuring on a Lunatic spectrophotometer. 2 µg (*A. thaliana*) of each sample was injected for LC–MS/MS analysis on an Ultimate 3000 RSLCnano system in-line connected to a Q Exactive HF mass spectrometer equipped with a Nanospray Flex Ion Source. Trapping was performed at 10 μl/min for 4 min in solvent A on a 20 mm trapping column (made in-house, 100 μm internal diameter (I.D.), 5 μm beads, C18 Reprosil-HD) and the plant sample was loaded on a 200 cm long micro-pillar array column with a C18-endcapped functionality mounted in the Ultimate 3000’s column oven at 50 °C. For proper ionization, a fused silica PicoTip emitter (10 µm I.D.) was connected to the µPAC™ outlet union and a grounded connection was provided to this union. Peptides were eluted by a non-linear increase from 1 to 55% MS solvent B (0.1% FA in water/ACN (2:8, v/v)) over 145 min, first at a flow rate of 750 nl/min, then at 300 nl/min, followed by a 15 min wash reaching 99% MS solvent B and re-equilibration with MS solvent A (0.1% FA in water). The mass spectrometer was operated in data-dependent mode, automatically switching between MS and MS/MS acquisition for the 16 most abundant ion peaks per MS spectrum. Full-scan MS spectra (375–1500 m/z) were acquired at a resolution of 60,000 in the Orbitrap analyzer after accumulation to a target value of 3E6. The 16 most intense ions above a threshold value of 1.3E4 (minimum AGC of 1E3) were isolated for fragmentation at a normalized collision energy of 28%. The C-trap was filled at a target value of 100,000 for maximum 80 ms and the MS/MS spectra (200–2000 m/z) were acquired at a resolution of 15,000 in the Orbitrap analyzer with a fixed first mass of 145 m/z. Only peptides with charge states ranging from + 2 to + 6 were included for fragmentation and the dynamic exclusion was set to 12 s.

### Identification and quantification of proteins

LC–MS/MS runs of all samples were searched together using the MaxQuant algorithm with mainly default search settings, including a false discovery rate set at 1% on PSM, peptide and protein level. Spectra were searched for *Saccharomyces cerevisiae* against the *Saccharomyces cerevisiae* protein sequences in the Uniprot database containing 6049 sequences and for *A. thaliana* against the *Arabidopsis* protein sequences in the Swiss-Prot database, containing 39,359 sequences. The mass tolerance for precursor and fragment ions was set to 4.5 and 20 ppm, respectively, during the main search. Enzyme specificity was set as C-terminal to arginine and lysine, also allowing cleavage at proline bonds with a maximum of two (*S. cerevisiae*)/three (*A. thaliana)* missed cleavages. Variable modifications were set to oxidation of methionine residues and acetylation of protein N-termini, while carbamidomethylation of cysteine residues was set as a fixed modification for *A. thaliana* samples. Matching between runs was enabled with a matching time window of 0.7 min and an alignment time window of 20 min. Only proteins with at least one unique or razor peptide were retained. Proteins were quantified by the MaxLFQ algorithm integrated in the MaxQuant software. A minimum ratio count of two unique or razor peptides was required for quantification.

To compare protein intensities in the 18S probes and scrambled probes samples, statistical testing for differences between the two group means was performed, using the R-package Limma (moderated t test). Missing protein intensity values were imputed by randomly sampling from a normal distribution centered around each sample’s noise level. Statistical significance for differential regulation was set at adjusted p-value < 0.01 and |log2FC|= 2. Since our to compare datasets have a large difference in protein intensities (scrambled group should be theoretically lacking proteins) iBAQ intensities were chosen over MaxLFQ intensities for quantification. To appoint proteins to be part of the interactome both a semi-quantitative as a quantitative method were used. If proteins were not detected in any of the non-cross-linked samples but present in 4 of the 5 replicates of the condition this protein was appointed to be an interaction partner of 18S rRNA in a semi-quantitative manner.

Further data analysis of the shotgun results of the study of the RBPome of *A. thaliana* was performed with the Perseus software and Limma package (moderated T-test) in R. Since the datasets we want to compare have a large difference in protein intensities (control group should be theoretically lacking proteins) iBAQ intensities were chosen over MaxLFQ intensities for quantification. To appoint proteins to be part of the RBPome both a semi-quantitative as a quantitative method was used. If proteins were not detected in any of the non-cross-linked samples but present in 3 of the 5 replicates of the condition this protein was appointed to be an RBP in a semi-quantitative manner^71^. For proteins with an iBAQ value in both the non-cross-linked control and cross-linked conditions a quantitative method was applied. Using Perseus, proteins yielding minimal 3 iBAQ values per 5 replicates were selected, log-transformed and the missing values were imputed with values drawn from a normal distribution. This modified dataset was used to perform the moderated t-test implemented in the R/Bioconductor package Limma. The p-values were corrected for multiple testing using the Benjamini–Hochberg test to calculate the adjusted p-value. Proteins with an adjusted p-value < 0.01 and a |log2FC| (CL/No-UV) > 1.5 were appointed to be true RNA binding proteins.

### CryoEM structures of yeast ribosome

In order to visualise the proteins identified using our here presented approach we used PDB entry: 3JJ7. The images were rendered using pymol. Every chain within the structure was inspected and labelled as being significantly enriched or not. The non-ribosomal Guanine nucleotide-binding protein subunit beta-like protein was removed during visualisation.

### Gene Ontology (GO) enrichment analysis for the study of the RBPome of *A. thaliana*

Protein IDs of the identified RBPs were used to perform GO-analysis using Panther. The reference *Arabidopsis* proteome was used to calculate enrichment by applying the Fisher’s exact test and Bonferroni corrected p-values were used to account for multiple testing. GO-analysis was performed for both Biological process (BP), Molecular function (MF) and cellular component (CC) and compared between the different conditions or subsets within the unique and overlapping protein IDs between different conditions or subsets within the unique and overlapping protein IDs between conditions.

## Supplementary Information

Supplementary Information.

## Data Availability

The datasets generated and/or analysed during the current study are available in the PRIDE repository, https://www.ebi.ac.uk/pride/archive/. For the 18S rRNA interactome, the mass spectrometry proteomics data have been deposited to the ProteomeXchange Consortium via the PRIDE [1] partner repository with the dataset identifier PXD031573. Username: reviewer_pxd031573@ebi.ac.uk. Password: nHKrsXU8. For the RBPome of *A. thaliana*, the mass spectrometry proteomics data have been deposited to the ProteomeXchange Consortium via the PRIDE [1] partner repository with the dataset identifier PXD031578. Username: reviewer_pxd031578@ebi.ac.uk. Password: bfOD8ODc.
