# Supplementary material for: A widely applicable and cost-effective method for specific RNA–protein complex isolation
Source: Sci Rep. 2023 Apr 27;13:6898. doi: 10.1038/s41598-023-34157-0 (PMC10140378; doi:10.1038/s41598-023-34157-0)
Supplement: Supplementary file 1 — Supplementary Information. [file 41598_2023_34157_MOESM1_ESM.pdf]

A widely applicable and cost-effective method for specific RNA-protein complex isolation

Sam Balzarini, Roosje Van Ende, Arnout Voet and Koen Geuten

Supplementary figures

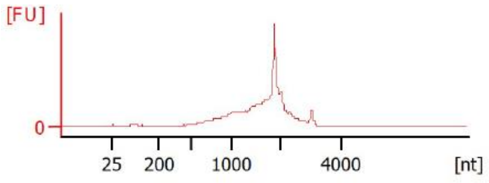

Supplementary figure S1. RNA pico BioAnalyzer (Agilent)
Capture with 18S probes (non-cross-linked sample).

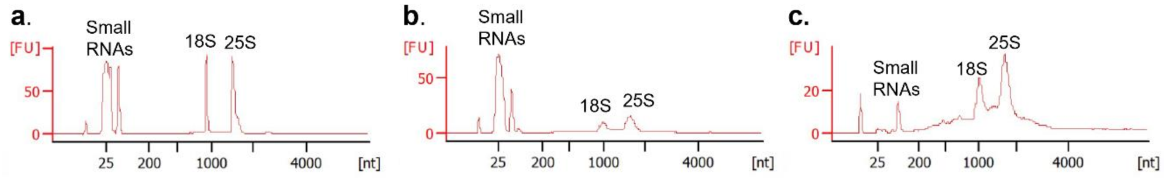

Supplementary figure S2. Decrease in RNA integrity due to UV cross-linking, *S. cerevisiae* a. non-crosslinked sample after TRAPP purification. b. UV cross-linked sample (1.2 J/cm^2) after TRAPP purification. c. UV cross-linked sample (1.2 J/cm^2) after SAPS purification. Unexpectedly small RNAs appeared to be more efficiently purified with the TRAPP protocol when the sample was cross-linked. Hypothetically due to the fact that the smaller complexes have relatively fewer protein-interaction partners and by consequence a smaller chance to be UV cross-linked. These non-cross-linked molecules potentially have greater affinities to the silica beads. This hypothesis is supported by the increased abundance of longer molecules after the SAPS protocol as a whole when only the cross-linked complexes remain.

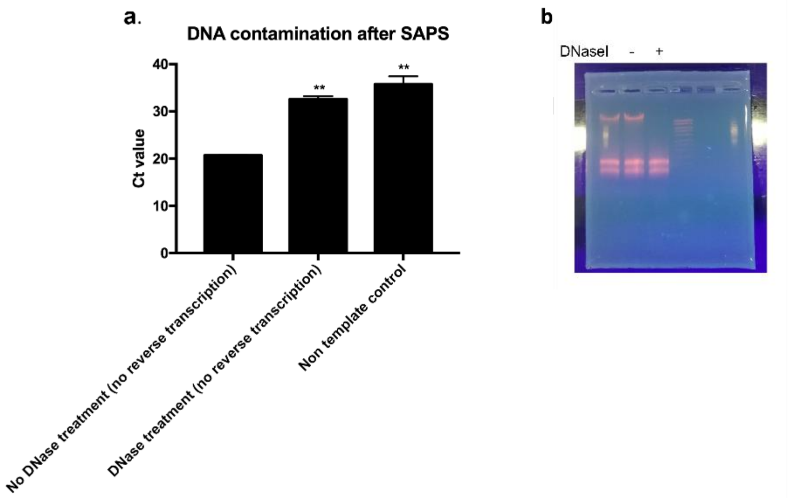

Supplementary figure S3. Presence of DNA contamination after TRAPP/SAPS, *S. cerevisiae*. a. Ct values of non-reverse transcribed samples of a SAPS purified sample with or without DNAse treatment. ** represents a two-tailed p-value<0.0, Error bars represent SEM, n=2. Significance is determined with an unpaired t-test. b. Agarose gel electrophoresis of a silica-based purification with or without DNase treatment, 1300ng, Chinese spring.

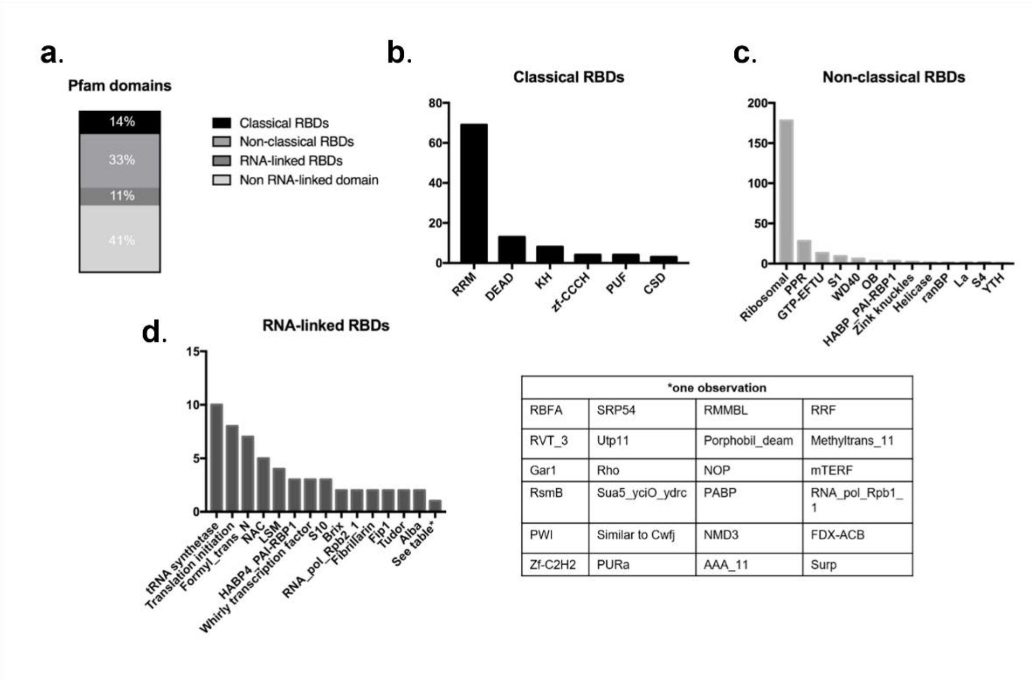

*one observation			
RBFA	SRP54	RMMBL	RRF
RVT_3	Utp11	Porphobil_deam	Methyltrans_11
Gar1	Rho	NOP	mTERF
RsmB	Sua5_yciO_ydrc	PABP	RNA_pol_Rpb1_1
PWI	Similar to Cwfj	NMD3	FDX-ACB
Zf-C2H2	PURa	AAA_11	Surp

Supplementary figure S4. Study of the RNA-binding domains of the SAPS isolated *A. thaliana* leaf RBPome a. the distribution of proteins harboring b. classical, c. non-classical, d. RNA-linked RBDs and domains not linked to RNA based on a Pfam annotation. For each RBD subset, the known RBD and the amount of proteins harboring this domain are represented.

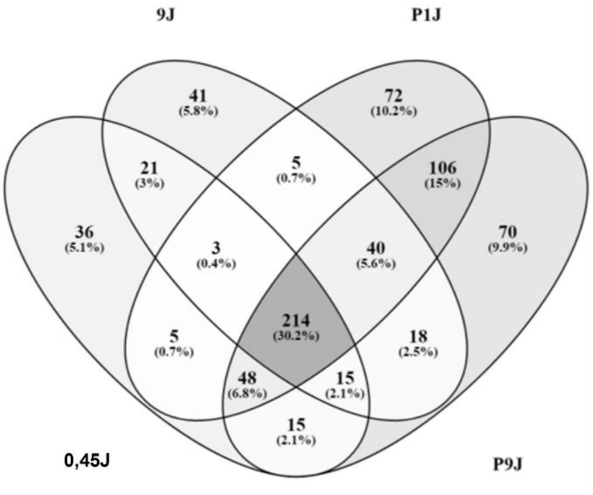

Supplementary figure S5. Comparison of occurring RBPs per UV cross-linking condition. The UV dose has been displayed in the figure where P stands for inducing UV light on frozen powder tissue instead of fresh leaf tissue.

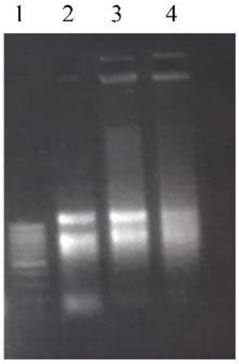

Supplementary figure S6. Agarose gel electrophoresis to analyse RNA integrity after UV cross-linking. Lane 1: marker, lane 2: non-cross-linked control, lane 3: 0.45J, lane 4: 9J

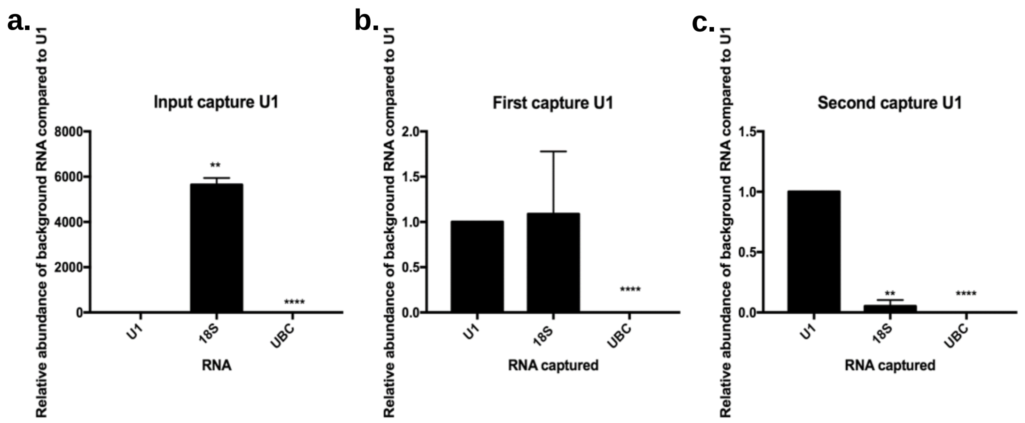

Supplementary figure S7. Capture of U1 in *S. cerevisiae* **** represents a two-tailed p-value<0.0001, ** a two-tailed p-value<0.01 a. Relative abundance of 18S/UBC compared to U1 after SAPS. Error bars represent SEM, n=2. b. Relative abundance of 18S/UBC compared to U1 after capture of U1 . Error bars represent SEM, n=2. c. Relative abundance of 18S/UBC compared to U1 after a second capture of U1 . Error bars represent SEM, n=2. Significance is determined with an unpaired t-test.

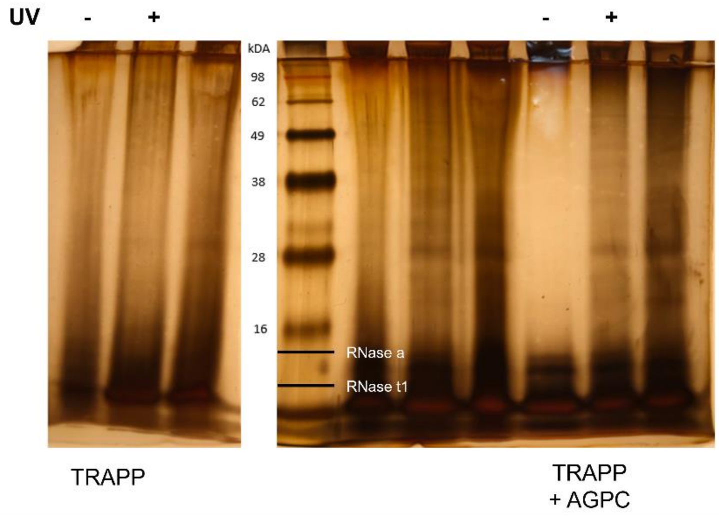

Supplementary figure S8. Full size figure 3

Supplementary tables

Supplementary table 1. Significantly enriched proteins of 18S rRNA interactome (small subunit=SSU, large subunit=LSU)

**Protein names**	**Gene names**	**Category**	**Adj. p-value**	**Log2 FC**
**Quantitative analysis** (sorted by adjusted P-value)				
40S ribosomal protein S5	RPS5	Protein SSU	5,85E-05	3,048589
40S ribosomal protein S6-B;40S ribosomal protein S6-A	RPS6B;RPS6A	Protein SSU	5,85E-05	2,64233
40S ribosomal protein S4-B;40S ribosomal protein S4-A	RPS4B;RPS4A	Protein SSU	5,85E-05	3,162362
40S ribosomal protein S8-B;40S ribosomal protein S8-A	RPS8B;RPS8A	Protein SSU	5,96E-05	2,580947
40S ribosomal protein S17-B;40S ribosomal protein S17-A	RPS17B;RPS17A	Protein SSU	5,96E-05	2,778226
Polyadenylate-binding protein, cytoplasmic and nuclear	PAB1	Potential link SSU	5,96E-05	2,873475
40S ribosomal protein S24-B;40S ribosomal protein S24-A	RPS24B;RPS24A	Protein SSU	5,96E-05	2,791031
40S ribosomal protein S2	RPS2	Protein SSU	5,96E-05	2,58491
40S ribosomal protein S26-B;40S ribosomal protein S26-A	RPS26B;RPS26A	Protein SSU	6,29E-05	2,273769
Nuclear and cytoplasmic polyadenylated RNA-binding protein PUB1	PUB1	Potential link SSU	6,29E-05	4,201088
40S ribosomal protein S11-B;40S ribosomal protein S11-A	RPS11B;RPS11A	Protein SSU	6,29E-05	2,93529
40S ribosomal protein S1-B	RPS1B	Protein SSU	6,29E-05	2,718774
Protein SCP160	SCP160	Potential link SSU	7,05E-05	4,151636
Elongation factor 3A;Elongation factor 3B	YEF3;HEF3	rRNA biogenesis	7,05E-05	3,738714
Nucleolar protein 3	NPL3	rRNA biogenesis	7,25E-05	2,334254
40S ribosomal protein S7-A	RPS7A	Protein SSU	7,70E-05	4,218388
	RPS9B;RPS9A	Protein SSU	7,70E-05	2,049898

40S ribosomal protein S9-B;40S ribosomal protein S9-A				
40S ribosomal protein S0-B;40S ribosomal protein S0-A	RPS0B;RPS0A	Protein SSU	7,70E-05	4,578979
60S ribosomal protein L24-B;60S ribosomal protein L24-A	RPL24B;RPL24A	Protein LSU	7,74E-05	2,564207
Pyruvate kinase 1;Pyruvate kinase 2	CDC19;PYK2	Without known ribosome-related association	0,000119	3,296296
40S ribosomal protein S13	RPS13	Protein SSU	0,000126	3,832276
Nuclear localization sequence-binding protein	NSR1	rRNA biogenesis	0,000161	3,870642
40S ribosomal protein S3	RPS3	Protein SSU	0,00018	2,385512
60S ribosomal protein L4-A;60S ribosomal protein L4-B	RPL4A;RPL4B	Protein LSU	0,000366	2,256553
40S ribosomal protein S19-B;40S ribosomal protein S19-A	RPS19B;RPS19A	Protein SSU	0,000509	3,703472
40S ribosomal protein S18-B;40S ribosomal protein S18-A	RPS18B;RPS18A	Protein SSU	0,000509	5,221024
Nuclear segregation protein BFR1	BFR1	Potential link SSU	0,000571	3,61529
Eukaryotic translation initiation factor 1A	TIF11	rRNA biogenesis	0,000601	3,883685
Eukaryotic translation initiation factor 3 subunit C	NIP1	rRNA biogenesis	0,000862	3,048179
60S ribosomal protein L3	RPL3	Protein LSU	0,001271	2,645415
Elongation factor 2	EFT1	rRNA biogenesis	0,001788	3,678304
60S ribosomal protein L10	RPL10	Protein LSU	0,002135	3,332925
Plasma membrane ATPase 1;Plasma membrane ATPase 2	PMA1;PMA2	Without known ribosome-related association	0,002745	4,715109

ATP-dependent RNA helicase eIF4A	TIF1	rRNA biogenesis	0,002745	3,107831
Single-stranded nucleic acid-binding protein	SBP1	Potential link SSU	0,003022	3,072121
60S ribosomal protein L19-B;60S ribosomal protein L19-A	RPL19B;RPL19A	Protein LSU	0,003074	2,750949
Glyceraldehyde-3-phosphate dehydrogenase 2;Glyceraldehyde-3-phosphate dehydrogenase 3;Glyceraldehyde-3-phosphate dehydrogenase 1	TDH2;TDH3;TDH1	Without known ribosome-related association	0,003162	2,533137
60S ribosomal protein L36-A;60S ribosomal protein L36-B	RPL36A;RPL36B	Protein LSU	0,00388	3,988627
Multiprotein-bridging factor 1	MBF1	Without known ribosome-related association	0,00487	2,654672
60S ribosomal protein L7-B;60S ribosomal protein L7-A	RPL7B;RPL7A	Protein LSU	0,005058	2,840046
Putative uncharacterized protein YCL042W	SGD:S000000547	Without known ribosome-related association	0,00566	3,209975
40S ribosomal protein S25-A;40S ribosomal protein S25-B	RPS25A;RPS25B	Protein SSU	0,00566	3,546065
**Semi-quantitative analysis** (sorted by abundance)				
40S ribosomal protein S20	RPS20	Protein SSU		
40S ribosomal protein S1-A	RPS1A	Protein SSU		
Nucleolar protein 58	NOP58	rRNA biogenesis		
60S ribosomal protein L34-A;60S ribosomal protein L34-B	RPL34A;RPL34B	Protein LSU		
60S ribosomal protein L30	RPL30	Protein LSU		

40S ribosomal protein S10-A;40S ribosomal protein S10-B	RPS10A;RPS10B	Protein SSU
60S ribosomal protein L15-A;60S ribosomal protein L15-B	RPL15A;RPL15B	Protein LSU
40S ribosomal protein S16-B;40S ribosomal protein S16-A	RPS16B;RPS16A	Protein SSU
40S ribosomal protein S23-B;40S ribosomal protein S23-A	RPS23B;RPS23A	Protein SSU
Ribosome biogenesis protein RLP7	RLP7	rRNA biogenesis
Nucleolar protein 56	NOP56	rRNA biogenesis
Phosphoglycerate kinase	PGK1	Without known ribosome-related association
Eukaryotic initiation factor 4F subunit p150	TIF4631	rRNA biogenesis

Supplementary table 2. Small ribosomal proteins sorted by length (Bolt: identified by MS)

**Protein**	**Length (aa)**
RPS29A, RPS29B	56
RPS30A, RPS30B	63
RPS28A, RPS28B	67
RPS27A, RPS27B	82
RPS21A, RPS21B	87
**RPS10A, RPS10B**	**105**
**RPS25A;RPS25B**	**108**
**RPS26B;RPS26A**	**119**
**RPS20**	**121**
RPS22	130
**RPS24B;RPS24A**	**135**
**RPS17B;RPS17A**	**136**
RPS14	137
RPS15	142
**RPS16A, RPS16B**	**143**
RPS12	143

**RPS19B;RPS19A**	**144**
**RPS23A, RPS23B**	**145**
**RPS18B;RPS18A**	**146**
**RPS13**	**151**
RPS31;RPL40B;RPL40A;UBI4	152
**RPS11B;RPS11A**	**156**
**RPS7A**	**190**
**RPS9B;RPS9A**	**197**
**RPS8B;RPS8A**	**200**
**RPS5**	**225**
**RPS6B;RPS6A**	**236**
**RPS3**	**240**
**RPS0B;RPS0A**	**252**
**RPS2**	**254**
**RPS1B**	**255**
**RPS4B;RPS4A**	**261**

Supplementary table 3. PCR and RT-qPCR primers

**PCR primers**	
PCR1_18sF	5’-aataagggttcgattccggag-3’
PCR2_18sR	5’-aaagggcagggacgtaatc-3’
**qPCR primers**	
qPCR1_18sF	5’-cctttccttctggctaaccttgagtccttg-3’
qPCR2_18sR	5’-cgagcaatacgcctgctttgaacactc-3’
qPCR3_25sF	5’-cccactgtccctatctactatc-3’
qPCR4_25SR	5’-gctcaacagggtcttctttc-3’
qPCR5_taf10F	5’-atattccaggatcaggtcttccgtagc-3’
qPCR6_taf10R	5’-gtagtcttctcattctgttgatgttgttgttg-3’

Supplementary table 4. Probes for RNP-targeting of 18S rRNA

**18S probes**
5’tacttagacatgcatggcttaatctttgagacaagcatatgactactggcaggatcaacc /3BioTEG/3’

5'cggtactagcgacgggcggtgtgtacaaagggcagggacgtaatcaacgcaagctgatga/3BioTEG/3'
5' ttaggattgggtaatttgcgcgcctgctgccttccttggatgtggtagccgtttctcagg /3BioTEG/3'
5'actcgctggctccgtcagtgtagcgcgcgtgcggcccagaacgtctaagggcatcacaga/3BioTEG/3'
5'ttaactgcaacaactttaatatacgctattggagctggaattaccgcggctgctggcacc /3BioTEG/3'
**Scrambled probes**
5'gtttcaacacgtttacgacacttttcaaggtgactacggccacaacaattatacacatca/3BioTeg/3'
5'tattagcgacacttcgatactgaaagggtatgtaccgtcacagaccggaacgccctcaga/3BioTeg/3'
5'cagtccgtgcgggtaattctgacctccagttcgaataactcctctcgaatagtcacgata/3BioTeg/3'
5'ttcggtagtactcttgcgatgatagagacctcctcggaactccagctatcctgtagatcg/3BioTeg/3'
5'tgagtgtgcgacacctcccaaaggaaggccgcccactcatctagcccaaataacctgtaa/3BioTeg/3'
